# Carbon Nanotubes—Potent Carriers for Targeted Drug Delivery in Rheumatoid Arthritis

**DOI:** 10.3390/pharmaceutics13040453

**Published:** 2021-03-27

**Authors:** Camilla Kofoed Andersen, Sangita Khatri, Jonas Hansen, Sofie Slott, Rohith Pavan Parvathaneni, Ana C. Mendes, Ioannis S. Chronakis, Shu-Chen Hung, Narendiran Rajasekaran, Zhuoran Ma, Shoujun Zhu, Hongjie Dai, Elizabeth D. Mellins, Kira Astakhova

**Affiliations:** 1 Department of Chemistry, Technical University of Denmark, Kemitorvet 206, 2800 Kongens Lyngby, Denmark; millaa@live.dk (C.K.A.); khatrisangita2049@gmail.com (S.K.); jonhan@kemi.dtu.dk (J.H.); sofslo@kemi.dtu.dk (S.S.); s181353@student.dtu.dk (R.P.P.); 2 DTU-Food, Technical University of Denmark, Kemitorvet 202, 2800 Kongens Lyngby, Denmark; anac@food.dtu.dk (A.C.M.); ioach@food.dtu.dk (I.S.C.); 3 Department of Pediatrics, Program in Immunology, Stanford University School of Medicine, Stanford, CA 94305, USA; shung01@amgen.com (S.-C.H.); mellins@stanford.edu (E.D.M.); 4 Department of Chemistry and Biochemistry, Northern Arizona University, Flagstaff, AZ 86004, USA; Naren.raj@nau.edu; 5 Department of Chemistry, Stanford University, Stanford, CA 94305, USA; zma2@stanford.edu (Z.M.); sjzhu@jlu.edu.cn (S.Z.); hdai1@stanford.edu (H.D.)

**Keywords:** carbon nanotubes, rheumatoid arthritis, siRNA

## Abstract

Two types of single-walled carbon nanotubes (SWCNTs), HiPco- and carboxyl-SWCNT, are evaluated as drug carriers for the traditional anti-inflammatory drug methotrexate (MTX) and a small interfering RNA (siRNA) targeting NOTCH1 gene. The nanotubes are solubilized by PEGylation and covalently loaded with MTX. The coupling efficiency (CE%) of MTX is 77–79% for HiPco-SWCNT and 71–83% for carboxyl-SWCNT. siRNA is noncovalently attached to the nanotubes with efficiency of 90–97% for HiPco-SWCNT and 87–98% for carboxyl-SWCNT. Through whole body imaging in the second near-infrared window (NIR-II window, 1000–1700 nm), SWCNTs were found to be selectively accumulated in inflamed joints in a serum transfer mouse model. We further investigated the interactions of the siRNA/MTX loaded nanotubes with human blood and mice bone marrow cells. In human blood, both types of unloaded SWCNTs were associated with B cells, monocytes and neutrophils. Interestingly, loading with MTX suppressed SWCNTs targeting specificity to immune cells, especially B cells; in contrast, loading siRNA alone enhanced the targeting specificity. Loading both MTX and siRNA to carboxyl-SWCNT enhanced targeting specificity to neutrophils and monocytes but not B cells. The targeting specificity of SWCNTs can potentially be adjusted by altering the ratio of MTX and siRNA loaded. The combined results show that carbon nanotubes have the potential for delivery of cargo drugs specifically to immune cells involved in rheumatoid arthritis.

## 1. Introduction

Rheumatoid arthritis (RA) is a chronic inflammatory autoimmune disease that affects 0.5–1% of the adult population worldwide, predominantly affecting women and the elderly [1]. Although the exact mechanism of RA development is still unknown, both susceptibility genetic factors and environmental factors are known to play significant roles [2]. RA is primarily characterized by joint swelling that reflects inflammation of synovial membrane, where leukocytes, such as neutrophils, monocytes, T cells, and B cells, are recruited to trigger onset of RA and lead to destruction of cartilage and bone [1,3]. The most commonly used therapies for managing RA are disease-modifying antirheumatic drugs (DMARDs) [4]. These drugs slow disease progression and can therefore potentially save joints and tissues from permanent damage. DMARDs work though several different mechanisms and can be classified as so-called synthetic or biological drugs [5,6]. Methotrexate (MTX), folic-acid antagonist, is a synthetic drug that is often used to initiate the treatment for RA [4,7]. However, MTX has toxicity and a significant portion of patients on MTX monotherapy or combination therapy have experienced gastrointestinal adverse events and hepatotoxicity, which leads to MTX withdrawal [8,9,10].

Efforts to reduce the MTX toxicity include the use of nanomaterials as delivery systems to specifically target cells or tissues [11]. These nanomaterials include dendrimers, lipid particles and carbon nanotubes (CNTs) [12,13,14]. Besides reducing toxicity, delivery systems also have the potential to increase drug half-life, reduce immunogenicity and improve bioavailability [15].

So far, CNTs have been primarily employed for applications in cancer treatment [16]. CNTs are easily functionalized by surface alterations through noncovalent and covalent attachment and, thus, can be applied for the delivery of both small and large molecules. Soluble CNTs functionalized by surface oxidization and coated by surfactants or amphiphilic polymers are able to be engulfed by cells via the energy-dependent endocytosis pathway. A few studies have demonstrated the successful use of CNTs in arthritis. Specifically, PEG polymers covalently attached to CNTs were used as an intra-articular delivery source for chondrocytes in osteoarthritis (OA)-induced mouse models; the coupling of pharmacological agents with an intra-articular delivery nanosystem enhanced drug residence time and increased cartilage penetration in OA joints [17].

Gene therapy represents another approach for RA treatment. Examples of in vivo studies include poly(lactic-co-glycolic acid (PLGA) nanoparticles loaded with siRNA used to target TNF-α (Tumor Necrosis Factor alpha) [18] and micelles loaded with siRNA and dexamethasone to target the transcription factor NF-κB [19]. An emerging target for potential gene therapy in RA is the Notch signaling pathway [20,21]. Expression and activation of Notch signaling has been observed in multiple cell types in RA synovium [22,23,24,25,26,27,28,29]. In addition, administration of a Notch inhibitor ameliorated arthritis in a collagen-induced arthritis model [24]. Adding to the complexity of Notch signaling in RA, another study using siRNA to target the γ-secretase necessary for NOTCH1 activation found that the suppressed autoimmune response was accompanied with an increase in regulatory T cells [25].

In this study, two different types of single wall carbon nanotubes (SWCNTs) were linked with siRNA or MTX, or both. The products were then evaluated for use as drug carriers. Attachment with SWCNTs has the potential to enhance efficacy and reduce the associated toxicity of MTX. The two types of SWCNTs used in this study are high-pressure carbon monoxide synthesized SWCNT (HiPco-SWCNTs) and COOH functionalized SWCNT (carboxyl-SWCNTs). Unmodified SWCNTs are highly hydrophobic and toxic. In order to use SWCNTs in vivo it is therefore necessary to functionalize their surfaces. SWCNTs are commonly functionalized with polyethylene glycol (PEG) as PEG provides SWCNTs with increased solubility, stability, and reduced toxicity [26,27], resulting in an overall increase in biocompatibility [28]. The synthesized conjugates in this study include solubilized SWCNTs loaded with MTX and/or an siRNA targeting NOTCH1 or scramble sequence. We first developed a synthetic strategy for these products and characterized them. Next, the solubilized nanotubes without any cargo were tested for toxicity in a serum transfer mouse model for RA [29]. The final SWCNTs were then tested for specific cell interaction in human blood and bone marrow. Additionally, their drug stability properties were evaluated.

## 2. Materials and Methods

### 2.1. Reagents

Raw Small Diameter SWCNTs (HiPco-SWCNTs) (≈3.4 × 10^5^–5.2 × 10^6^ g/mol) were purchased from NanoIntegris (Boisbriand, QC, Canada). Short COOH Functionalized Single Walled-Double Walled Carbon Nanotubes (carboxyl-SWCNTs) 1–4 nm were purchased from Cheap Tubes Inc. (Grafton, VT, USA). DSPE-PEG-NH2 (5000 Da) and mPEG-DSPE (5000 Da) were purchased from Laysan Bio Inc. (Arab, AL, USA). All other reagents were of analytical grade and purchased from Sigma–Aldrich (Munich, Germany).

### 2.2. siRNA Synthesis

Oligonucleotides were synthesized using standard (Bz-A-CE, Ac-C-CE, Ac-G-CE, U-CE) and modified (2′-O-Me-U-CE) phosphoramidites from Glen Research. Solid-phase synthesis conditions using Biosset ASM-800ET DNA/RNA Synthesizer with reagents purchased from Sigma Aldrich: TCA Deblock, DCI activator 0.25 M, Oxidizer 0.02 M, Cap A and Cap B. The phosphoramidites were all prepared in 0.07 M solutions using dry acetonitrile and oligonucleotides synthesized on 1 µmol scale using universal solid support 1000 Å (CPG 1000 Å) from Sigma Aldrich. Oligonucleotides were cleaved from solid support using methylamine solution (33 wt% in absolute ethanol) at 65 °C for 2 h, desilylated with triethylamine trihydrofluorid in dry triethylamine and dry N-methyl-2-pyrrolidone and precipitated from cold acetone. The identity of oligonucleotides was established by mass spectrometry (MS) using an Autoflex speed MALDI-TOF mass spectrometer (Bruker Daltonics, Bremen, Germany). The oligonucleotides were co-spotted with 3-hydroxypicolinic acid as matrix on an MTP AnchorChip target plate for the analysis. The obtained mass spectra were recorded by the flexControl 3.4 (Bruker Daltonics, Bremen, Germany) software. The oligonucleotides were purified on a Ultimate 3000 UHPLC (Dionex, Sunnyvale, CA, USA) using a DNA-Pac RP (Thermo Fisher Scientific, Waltham, MA, USA) column (4 µm, 3.0 × 100 mm^2^) with a gradient of 5–15% buffer B in A over 30 min at 60 °C. (buffer A: 0.05 M TEAA, buffer B: 25% A in acetonitrile). Peaks were monitored at 260 nm.

### 2.3. Solubilization of Nanotubes

For full experimental description of nanoparticle synthesis, see Supporting Information. PEGylating of HiPco-SWCNTs and carboxyl-SWCNTs was achieved according to literature procedure [30,31] using a mixture of DSPE-PEG-NH_2_ and mPEG-DSPE in large excess. Briefly, the nanotubes (0.6 mg/mL) were suspended in an aqueous solution of sodium cholate (15 mg/mL) by vortexing and sonication. A solution of PEG in minimal volume of DMSO was then added and the mixture was further sonicated (molar ratio for HiPco:mPEG-DSPE:DSPE-PEG-NH_2_ was 1:1846:754), (mass ratio for SWCNTs:mPEG-DSPE:DSPE-PEG-NH_2_ was 1:1.78:0.73). Following centrifugation, the supernatant containing the solubilized nanotube was decanted and reduced. The product was then filtered by 30 kDa Amicon size exclusion filter and reduced to dryness for further use.

### 2.4. UV-Vis to Estimate DSPE-PEG-NH_2_ Loading

Fluorescamine assay was carried out using a serial dilution of the solubilized nanotubes as described [30]. Encapsulation efficiency (EE%) was calculated using the DSPE-PEG-NH_2_ calibration curve (see Supplemental Figure S3) and obtained using fluorescence emission values at 465 nm. EE% for DSPE-PEG-NH_2_ was found to be 52% for HiPco-SWNT and 63–80% for carboxyl-SWCNTs.

### 2.5. MTX Coupling

The covalent coupling of MTX to solubilized nanotubes was achieved by EDC/NHS chemistry in aqueous bicarbonate buffer (1 µM, pH 8.4). The amounts used of MTX and coupling reagents were based on the fluorescamine assay, as described in Section 2.4. MTX and coupling reagents were allowed to react for 1.5 h before addition of solubilized nanotubes (molar ratio for DSPE-PEG-NH_2_:NHS:EDC:MTX was 1:8:8:3.4). After reaction overnight at 5 °C, the product was filtered by 30 kDa Amicon size exclusion filter and reduced to dryness for further use. The coupling efficiency of MTX was determined using UV-Vis spectroscopy measuring the absorbance at 303 nm. The coupling efficiency in percent was calculated using the following equation, where *w_r_* represents the mass of the drug remaining in solution and *w_t_* represents the total mass of the drug added to the solution:

$$CE\%=\;\left(\frac{w_{t}-w_{r}}{w_{t}}\right)\times 10\%$$

### 2.6. PEI Coupling to Carboxyl-SWCNTs

The covalent coupling of polyethyleneimine (PEI) to carboxyl-SWCNTs was achieved by EDC/NHS chemistry in aqueous bicarbonate buffer (1 µM, pH 8.4). Carboxyl-SWCNTs and coupling reagents were allowed to react for 1.5 h before addition of PEI (molar ratio carboxyl-SWCNTs:NHS:EDC:PEI was 1:0.04:0.026:0.0000096). After reaction overnight at 5 °C, the product was filtered by 30 kDa Amicon size exclusion filter and reduce to dryness for further use.

### 2.7. Cy5.5 Conjugation to SWCNTs

Cy5.5 NHS-ester reagent (Lumiprobe) was added to PEG-amine nanotubes in 10-fold molar excess, 100 mM bicarbonate buffer, pH 8.3. The mixture was kept rotating in the dark for 12 h and purified by 3 kDa Amicon size exclusion filter. Efficiency of labeling was estimated by measuring fluorescence emission at 630 nm and was 96%.

### 2.8. siRNA Attachment

Prior to use the siRNA was annealed by incubating equimolar amounts of sense and antisense strand in a PBS buffer solution (100 µL, 0.5 µM, pH 7.4). The suspension was then incubated in a PCR tube on a SimpliAmp™ Thermal Cycler for 10 min at 85 °C and thereafter cooled to rt over 30 min. The concentration of annealed duplex was measured on a QIAGEN QIAxpert at 260 nm. 0.05 eq. Duplex in water was then added to nanotube conjugate based on the amount of DSPE-PEG-NH_2_ in the product. The mixture was shaken at rt for 30 min followed by overnight at 5 °C. The final product was then filtered by 30 kDa Amicon size exclusion filter and dissolved in DEPC-treated (pyrogen-free) water. The attachment efficiency was estimated by measuring the amount of nonattached siRNA.

### 2.9. Nanotube Conjugates Incubation with Human Blood

Human heparinized venous blood samples were collected anonymously and obtained from the Stanford Blood Center. It was confirmed by the Stanford University IRB on the 16 January 2019, case no. 49485, to Prof. ED Mellins that individual investigator protocols are not needed for use of fully de-identified samples obtained from the Blood Center for the conducted assays.

Whole blood (250 µL) was aliquoted into 5 mL round-bottom polypropylene tubes. Then, 250 µL (20 μM) of nanotube conjugates diluted to desired concentration in RPMI 1640 was then added to the blood containing tubes for a final volume of 500 µL. The mixture was then incubated for 30 min at 37 °C followed by washes with 2 mL RPMI 1640 twice before proceeding to flow cytometry staining.

### 2.10. In Vivo Study in Mice

**Figure 1:**
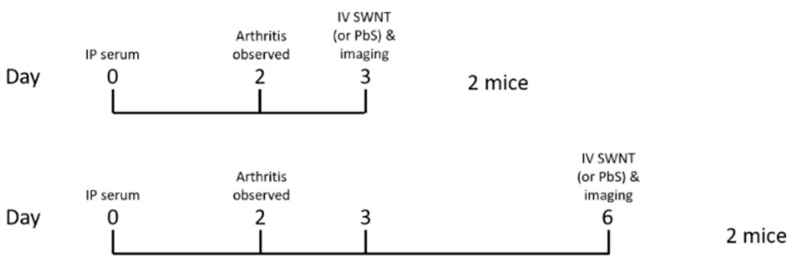
In vivo study—overview of procedures.

The animal study was performed under the approval of Stanford Institutional Animal Care and Use Committee (IACUC); the animal protocol number is APLAC-15867. Four Balb/c mice were intraperitoneally injected with K/BxN serum [27] on day 0, and the arthritis developed by day 3 and lasted for 3 days to reach the peak (see Methods, Figure 1). Two mice were intravenously injected with untagged HiPco-SWCNTs (50 µg) or lead sulfide/cadmium sulfide core/shell quantum dots (PbS) (0.5 mg) on day 3, and the other two mice were intravenously injected with HiPco-SWCNTs (50 µg) or PbS (0.5 mg) on day 6. Fluorescence images in the second near-infrared window (NIR-II window, 1000–1700 nm) were recorded using a liquid nitrogen-cooled two-dimensional InGaAs array (Teledyne Princeton Instruments, Trenton, NJ, USA) [29]. The excitation light was provided by a fiber-coupled 808-nm diode laser (RPMC Lasers) at a power density of 70 mW/cm^2^. The emission light was allowed to pass through a 910-nm long-pass filter combined with either a 1100-nm long-pass filter (for HiPco-SWCNTs) or a 1500-nm long-pass filter (for PbS). A set of achromatic lenses (focal length = 75 mm and 200 mm, respectively) was applied to focus the image onto the camera with a field of view of 62.5 × 50 mm^2^ (1x magnification) or 25 × 20 mm^2^ (2.5× magnification). NIR-II fluorescence images were collected with LabView software with an exposure time of 150 ms (HiPco-SWCNTs) or 50 ms (PbS). The images were processed with MATLAB (MathWorks, Natick, MA, USA).

### 2.11. Flow Cytometry Staining and Analysis in Human Blood

The procedure was conducted as previously described [32] with slight modification. Briefly, the blood cell pellet was stained with LIVE/DEAD Aqua for 10 min at rt followed by washing with 500 µL flow buffer. Cells were stained with antibody cocktails with the following fluorochrome-conjugated antibodies for 30 min at rt: Alexa Fluor 488-labeled anti-CD20 (clone 2H7); FITC-labeled anti-CD56 (clone HCD56); Pacific Blue-labeled anti-CD15 (clone W6D3); APC-labeled anti-CD3 (clone UCHT1); Brilliant Violet 785-labeled anti-CD14 (clone M5E2); Brilliant Violet 605-labeled anti-CD11b (clone ICRF44). BD lysing solution was then added to lyse red blood cells and fix other nucleated cells for 30 min at rt. Cells were washed with wash buffer (PBS with 0.1% NaN_3_) twice and resuspended in 200µL flow buffer. Cells were analyzed on a Becton Dickinson LSRII Analyzer at Stanford Shared FACS facility. Data were analyzed using FlowJo version 10 (FlowJo LLC, Ashland, OR, USA).

### 2.12. SEM Studies

The morphology and size of the nanoparticles was analyzed by SEM. Nanotube products stored in distilled water were transferred to metal stubs with double-sided adhesive carbon tape and allowed to dry at rt in a fume hood. The products were further sputter-coated with a 6 nm layer of gold (Leica Coater ACE 200, Leica, Vienna, Austria) prior to their imaging in a Quanta FEG 3D (FEI, Eindhoven, The Netherlands) scanning electron microscope. The diameters of the nanoparticle products were measured using the SEM images through the image visualization software ImageJ (National Institutes of Health, Bethesda, MD, USA) [33,34].

### 2.13. In Vitro Studies

Serum stability test of SWCNT products and of siRNA controls was performed on two nanoparticle products in serum. Then, 20 µL (23 mg/mL) nanoparticles loaded with siRNA (0.75 µg/µL) and the same concentration of naked siRNA were added to human serum (90% in HBBS buffer, pH = 7.4) in total volume of 158 µL in an Eppendorf tube. The samples were mixed thoroughly and aliquoted equally in PCR tube and incubated at 37 °C. Aliquots were withdrawn at the following time points: 0 min, 30 min, 1 h, 2 h, 4 h, 8 h, 24 h, and 48 h. The aliquots were incubated with 50% DMSO at 70 °C for 1 h and analyzed by amicon 30 kDa following manufacturer’s protocol at 50 °C. The released siRNA was quantified in the wash by a Qiagen expert. Then, the samples were resolved in 10% standard denaturing polyacrylamide gels (8 M urea, 1 × TBE) with TBE buffer for 1 h at 100 V in Bio-Rad gel electrophoresis chamber. The gel was stained overnight with 1X gel red buffer and analyzed by Gel Doc EZ imager and image lab software.

## 3. Results

SWCNTs linked with siRNA or MTX or both were evaluated and a total of 12 conjugates (C1–C12) were synthesized as shown in Table 1. The synthesis approach for the 12 conjugates is shown in the Supplemental Figure S1. Initially the SWCNTs were solubilized, and then covalently conjugated with MTX. At the last step, siRNA was added, to form a noncovalent product with the nanotubes. The details on synthetic procedures can be found in the Supplemental Information.

### 3.1. PEGylating of Carbon Nanotubes

The HiPco-SWCNTs and carboxyl-SWCNTs were successfully solubilized by noncovalent conjugation to DSPE-PEG-NH_2_ and mPEG-DSPE. UV-Vis spectroscopy at 808 nm was used to determine the yield of PEGylation [35,36]. The concentration of HiPco-DSPE-PEG-NH_2_ (C1–C6) and SWCNTs-DSPE-PEG-NH_2_ (C7–C12) in the product solutions were found to be 187 µM (79% yield) and 122 µM (86% yield), respectively. Additionally, the encapsulation efficiency (EE%) for DSPE-PEG-NH_2_ was measured by a fluorescamine assay and found to be 52% for HiPco-SWCNTs and 63–80% for carboxyl-SWCNTs; see Supplemental Figure S3 for the calibration curve.

### 3.2. MTX Loading of Carbon Nanotubes

The covalent conjugation of MTX was performed on the solubilized nanotubes (C1, C3, C5, C7, C9, and C11). The coupling CE% for the PEGylation was used to calculate the necessary reagents. The CE% for MTX was determined by measuring the amount of MTX that was not conjugated; see Supplemental Figure S2 for the MTX calibration curve. The CE% was found to be 77–79% for HiPco-SWCNTs and 71–83% for carboxyl-SWCNTs (Table 1).

### 3.3. siRNA Attachment

Four oligonucleotides were synthesized with great purity (>90% by HPLC; Table 2) and annealed to form two pairs of siRNA. See Supplemental Figure S4 for HPLC and MALDI data on the prepared RNA. The sequences were similar to those previously used for gene knockdown of NOTCH1 [25]. In addition, we designed scrambled control siRNA. Noncovalent attachment was then performed on the solubilized nanotubes loaded with or without MTX. The efficiency for siRNA attachment was determined by measuring the amount of siRNA that did not get attached. The efficiency was found to be 90–97% for HiPco-SWCNTs and 87–98% for carboxyl-SWCNTs (Table 1).

### 3.4. Study in Mice

**Figure 2:**
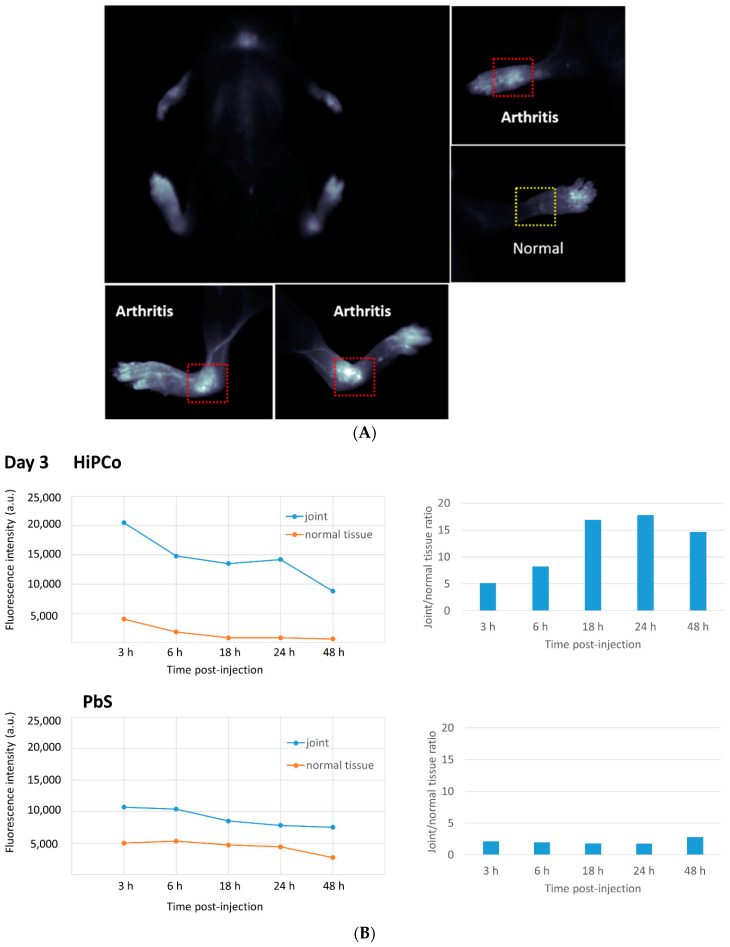
(**A**) Body image of mice 6–9 h after injection. Accumulation of HiPco single-walled carbon nanotubes (SWNTs) Cy5.5 in arthritis joints. (**B**) Relative fluorescence intensity of HiPco and lead sulfide/cadmium sulfide core/shell quantum dots (PbS) in joint and normal tissue (*left*); ratio of fluorescence intensity between joint and normal tissue for HiPco and PbS (*right*).

Solubilized HiPco-SWCNTs were labeled with cyanine5.5 NHS ester (Cy5.5) using the manufacturer’s protocol and tested for biodistribution in a serum-transfer arthritis mouse model (see Section 2.11). Near-infrared imaging showed the deposition of HiPco-SWCNTs Cy5.5 in arthritic joints starting at 2 h post i.v. injection and lasting at least until 48 h post-injection. In healthy control mice, only minimal HiPco-SWCNTs Cy5.5 was observed 4 h post injection (see Supplemental Figures S6 and S7). To determine whether the localization of SWCNT to arthritic joints was specific to SWCNT, non-targeting PbS quantum dots were compared to SWCNT in the RA mouse model. The whole-body imaging of arthritic mice showed specific deposition of HiPco-SWCNTs Cy5.5 in joints (Figure 2A and Supplemental Figure S8), whereas PbS deposited mainly in liver and spleen (Supplemental Figure S11, (a) and (c) data reported in [37]). In addition, the joint-targeting specificity of HiPco-SWCNTs Cy5.5 peaked at 24 h post injection (Figure 2B). The specific targeting of SWCNTs to inflamed tissue suggests a potential drug delivery system with high specificity and potency.

The higher fluorescence intensity after 3 and 6 h could be due to systemic circulation of HiPco-SWCNTs Cy5.5 conjugates. This effect was also observed for the surrounding tissue (data not shown). Compared with PbS, there is a clear enhancement of accumulation in the joint for HiPco-SWCNTs Cy5.5. PbS demonstrates a more static ratio between joint and surrounding tissue, which suggests less specific accumulation in the joint (Figure 2B).

In the second part of the in vivo study, we injected HiPco SWCNTs Cy5.5 and PbS to arthritic mice at day 6 (Figure 1, Methods). Whole body imaging of the mice at 24 h post injection showed specific accumulation of HiPco-SWCNTs Cy5.5 in joints (see Supplemental Figure S12) whereas there was no accumulation of PbS nanoparticles in joints (see Supplemental Figure S13). Fluorescence imaging of mice at day 6 showed a similar pattern to that at day 3 (see Supplemental Figures S14–S20), which indicates the better targeting of HiPco-SWCNTs Cy5.5 to arthritic joints than PbS nanoparticles.

### 3.5. SWCNT Study in Human Whole Blood

With the joint accumulation established by the in vivo data for HiPco-SWCNTs, we proceeded with testing drug-loaded variants. We aimed to determine whether functionalized nanotubes can specifically target any immune cells and therefore function as a drug carrier for RA treatment. We synthesized solubilized nanotubes loaded with MTX or siRNA, or both (Table 1). Human whole blood was incubated with products C1–C12 for 30 min at 37 °C followed by flow cytometry analysis (FACS; see Section 2.11). The obtained flow cytometric data is summarized in Figure 2 and shows that both types of PEGylated nanotubes are taken up by circulating B cells, monocytes and neutrophils in a dose-dependent manner (Hipco-SWCNTs for 1–6 and carboxyl-SWCNTs for 7–12; Figure 2). Notably, nearly 60% of B cells take up the nanotube products compared to 35~40% of monocytes at 400 nM.

In general, carboxyl-SWCNTs attached to siNOTCH interact more actively with B cells, neutrophils and monocytes compared to HiPco-SWCNTs (Figure 2, entries 7 and 8 compared to entries 1 and 2). However, this difference is not observed when scrambled siRNA is attached (Figure 2, entries 4 and 10).

For both HiPco- and carboxyl-SWCNTs, the presence of siRNA generally increases the interaction with cells, most likely due to charge mediated interactions [11], although HiPco-SWCNT attached to siNOTCH showed reduced interaction with cells (Figure 2, entries 2 and 6). B cells, neutrophils and monocytes are those most actively targeted (Figure 2, entries 1–4 and 7–10).

**Figure 3:**
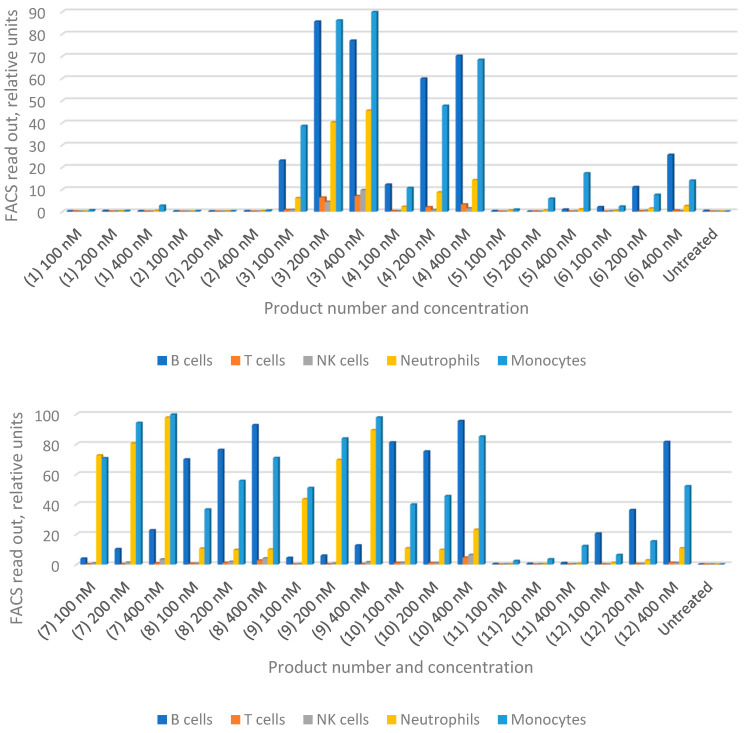
Flow cytometry analysis (FACS) data for nanotube products (C1–C12) incubated with human whole blood. See content of each product in Table 1. PEGylated indirect nanotubes HiPco-SWCNTs with and without MTX attached to siNOTCH (1 and 2) or scrambled siRNA (3 and 4); loaded with MTX (5); with no cargo in (6). PEGylated carboxyl-SWCNTs were with and without MTX attached to siNOTCH (7 and 8), or scrambled siRNA (9 and 10); loaded with MTX (11), with no cargo in (12).

Interestingly, conjugation of MTX modulates the targeting profile of the nanotube products as well. MTX suppressed nanotube uptake, especially in B cells (Figure 2, entries 5 and 11). However, in the presence of siRNA, MTX enhanced direct nanotube uptake by monocyte and neutrophil (Figure 3, entries 7–10). On the other hand, attachment of scrambled siRNA to indirect nanotubes not only slightly enhanced monocyte and neutrophil uptake but also reversed the suppressive effect of MTX on B cell uptake (Figure 2, entries 3 and 4). Notably, attachment of NOTCH1 siRNA almost completely suppressed cellular uptake of indirect nanotube (Figure 3, entries 1 and 2).

### 3.6. Bone Marrow Study

**Figure 4:**
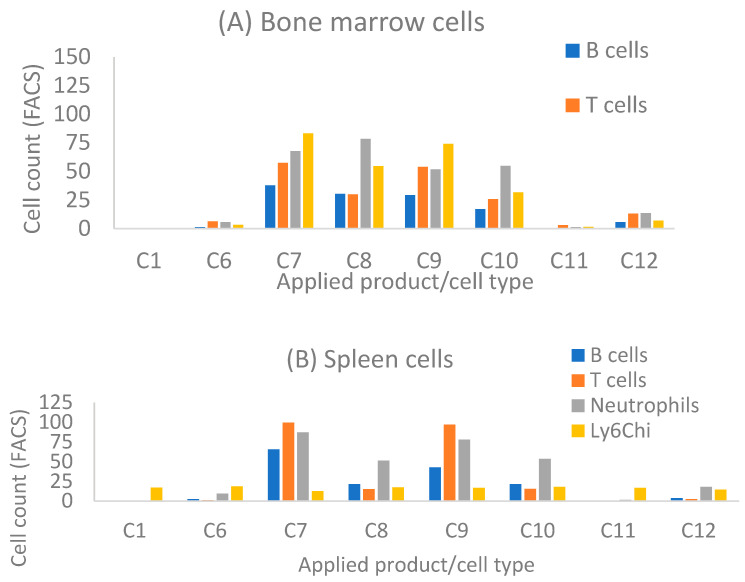
FACS results for nanotube products taken up by particular mouse immune cells in bone marrow (BM) (**A**), and spleen (**B**). BM and splenocytes were isolated from B6 mice and incubated with designated SWCNTs at 200 nM for 30 min. See details in Section 2. Applied products were as follows: Exchange (HiPco SWCNTs), 1. siNOTCH/MTX; 6. Not loaded nanotubes; Direct (carboxyl-SWCNTs), 7. siNOTCH/MTX, 8. siNOTCH, 9. siRNA/MTX, 10. siRNA, 11. MTX, 12. Not loaded nanotubes.

As near infrared imaging showed the specific deposition of nanotubes in arthritic joints, we further determined whether nanotube products can be taken up by particular mouse immune cells. Mouse bone marrow (BM) and splenocytes were isolated from B6 mice and incubated with designated nanotube product at 200 nM for 30 min. Flow cytometric analysis showed that both types of empty SWCNTs can be taken up by a small percentage of immune cells, including B cells, T cells, neutrophils, and the Ly6C^hi^ monocyte subset in BM and splenocytes (Figure 4). HiPco SWCNTs are taken up by fewer cells than carboxyl-SWCNTs, both in spleen and bone marrow (C1 and C6 data, Figure 3A,B). Similar to incubation with human blood (Figure 2), loading of MTX suppressed the uptake of carboxyl-SWCNTs (Figure 4A,B; data for C11). However, attachment of siRNA enhanced unconjugated or MTX-conjugated carboxyl-SWCNT uptake, except by splenic Ly6C^hi^ monocytes. Monocytes in BM are less mature and carry higher differentiation potential than peripheral (including splenic) monocytes. In addition, monocytes can differentiate into osteoclasts. The observed BM Ly6C^hi^ monocytes taking up carboxyl-SWCNTs suggest a potential for carboxyl-SWCNTs to disrupt osteoclast differentiation by blocking specific signaling pathways.

### 3.7. In Vitro siRNA Stability and SEM

**Figure 5:**
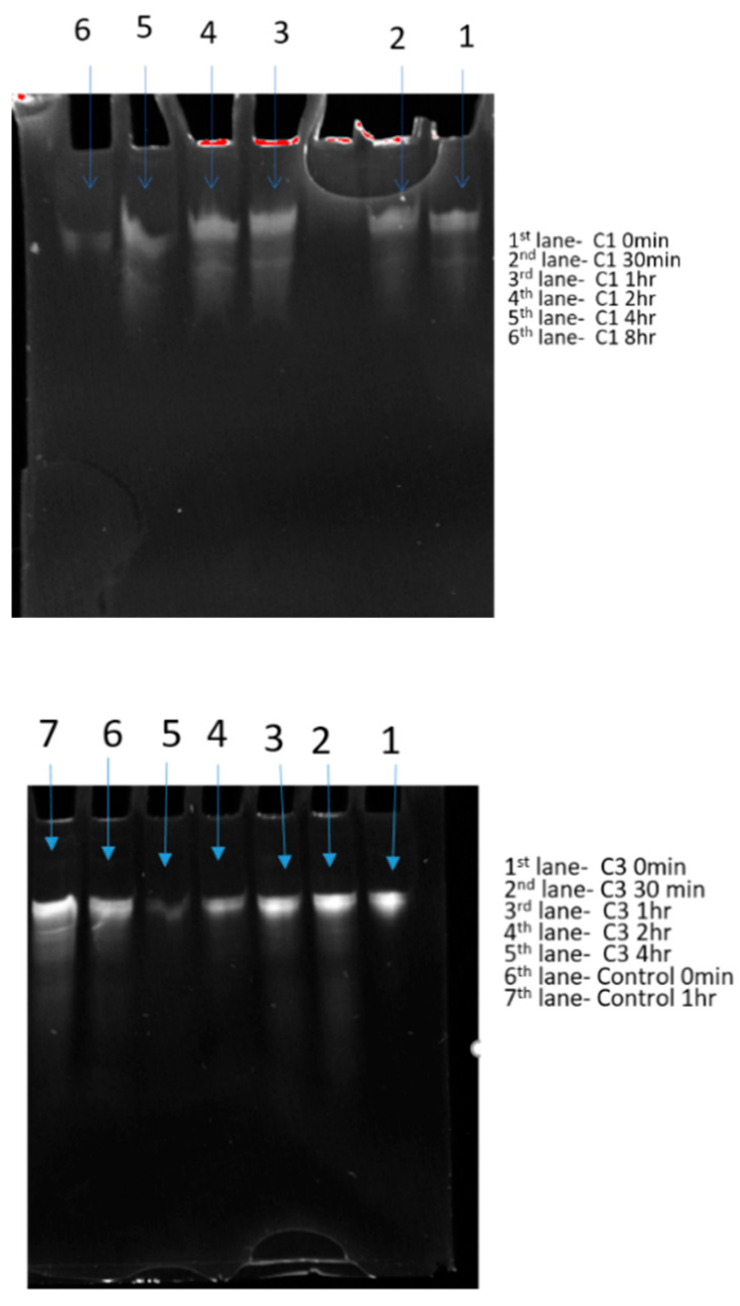
Gel images of siRNA obtained after in vitro stability study.

A serum stability test was performed on two selected nanotube products to evaluate their potential for in vivo studies. The siRNA-nanotubes and naked siRNA were incubated in 90% human sera for 0 min to 48 h with aliquots taken to determine the degradation. Notably, 90% and 85% of siRNA (Table 3) was still intact and could be released from nanoparticles C1 and C3, respectively, at time point 0. The intact siRNA decreased significantly over a period of 8 h for product C1 and 4 h for C3, whereas the naked siRNA was not detectable after 1 h (Figure 5). The decreased release of siRNA from nanoparticles might indicate the partial degradation of siRNA over period of 8 h and complete degradation after 24 h in human serum. However, the attachment of siRNA to nanoparticles improved the stability of siRNA in comparison to the naked siRNA control. Furthermore, these 13 selected samples were resolved in 10% standard denaturing polyacrylamide gels (8 M urea, 1 × TBE) with TBE buffer. Intact siRNA can be detected over a period of 8 and 4 h for nanoparticle products, whereas the control naked siRNA rapidly degrades in human serum. However, enzymatic bias from the products could be a factor as well. The intensity of the gel bands of siRNA decreases over time and follows the study table, indicating increased stability of siRNA by the products which demonstrates their potential as drug delivery vehicles (Figure 5).

**Figure 6:**
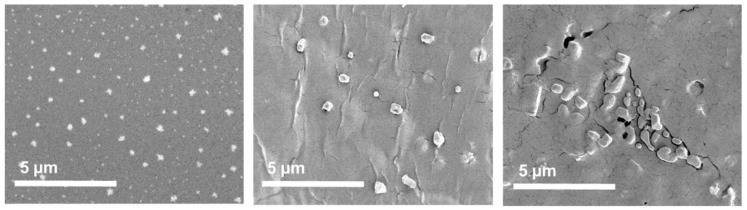
SEM images of HiPco-SWCNT: (**left**) to (**right**) solubilized HiPco-SWCNTs, solubilized HiPco-SWCNT attached to siRNA and MTX (C1), solubilized HiPco-SWCNT attached to scRNA and MTX (C3).

We studied solubilized HiPco-SWCNTs and HiPco-SWCNTs by scanning electron microscopy (SEM; Figure 6). Solubilized HiPco-SWCNTs displayed a regular spherical morphology (Figure 6) with an average diameter of 343.5 ± 42.58 nm (Table 4) and a very narrow size distribution (polydispersity index (PDI) 0.015).

The noncovalent attachment of siRNA and covalent linking to MTX (C1) increased the size of the particles to 518.8 ± 114.64 nm. The morphology became less spherical and more irregular in size distribution (PDI 0.049) resembling changes previously reported for MTX conjugates [38].

When attaching sc siRNA and MTX instead (C3), the morphology of the nanoparticles was similarly affected (Figure 6). Herein, agglomerated particles with irregular morphologies were produced with average size diameter of 407.67 ± 120.21 nm and slightly broader size distribution (PDI 0.087).

## 4. Discussion

In this work, our goal was to compare biological properties of two classes of carbon nanotubes, HiPco-SWCNTs and carboxyl-SWCNTs, as drug carriers for the standard of care drug, MTX, and a biological therapeutic candidate, anti-NOTCH1 siRNA. First, the six solubilized HiPco-SWCNTs were synthesized without any cargo drug and their pharmacokinetic distribution was determined by an in vivo fluorescence study. The PEGylated nanotubes were found to selectively accumulate in the joints of arthritic mice and not in healthy mice. Carbon nanotubes have been tested for delivery in arthritis models previously with less significant accumulation in joints [39]. The difference may be the solubilization by PEGylation, which was not performed in the previous example.

Other long-circulating nanoparticles, such as dextran-coated and others, are predominantly internalized by tissue macrophages (in lymph nodes, liver, spleen, bone marrow) and in circulating monocytes. Nanocarriers such as those prepared from lead sulfide (PbS) show nonspecific uptake but are prone towards uptake by immune system cells, including monocytes, macrophages and dendritic cells [40]. As an alternative, lipids are considered as promising drug carriers, which, however, often show limited stability in vivo and poor tissue specificity [41].

Inspired by these data, we synthesized and characterized 12 products based on HiPco-SWCNTs and carboxyl-SWCNTs. We compared these two classes of carbon nanotubes in terms of attachment properties for selected drugs, MTX and anti-NOTCH1 siRNA, and in terms of cellular interactions of products. The synthesis was characterized at each step to determine attachment efficiencies of the various components. For the HiPco-SWCNTs, we observed a noncovalent PEGylation of 52%, which is comparable to a reported example of 68% [27]. MTX was covalently conjugated through an amide bond, which has precedent in literature [42,43]. The amide bond to MTX is cleavable by lysosomal enzymes [41]. The CE% for MTX was found to be 77–79% for HiPco-SWCNTs and 71–83% for carboxyl-SWCNTs. CE% for MTX was not estimated in the literature examples.

siRNA was noncovalently attached to the nanotubes, and the efficiency was measured indirectly. The efficiency for noncovalent attachment was found to be 90–97% for HiPco-SWCNTs and 87–98% for carboxyl-SWCNTs. This is a rather high efficiency that points to suitability of the selected nanotube carriers for RNA drug delivery [44]. Besides natural RNA, modified variants with improved biological properties, such as locked nucleic acids (LNA) and 2′-OMe, can be applied in the future [44,45,46,47].

Targeted delivery is a critical feature to improve performance of conventional and biological drugs in diseases like RA. Moreover, toxicity of labeled HiPco-SWCNT in inflamed joints and not in the normal tissue is an advantageous property of the developed delivery system and is superior to poor tissue discrimination reported for e.g., lipids and PbS [37,40,41].

To the best of our knowledge, detailed studies of interactions for blood cells with MTX/RNA loaded carbon nanotubes are lacking. Interactions with blood cells are however critical for potential in vivo applications, when the nanotubes enter the blood stream. We conducted FACS studies on PEGylated HiPco-SWCNTs and carboxyl-SWCNTs. These studies showed that both classes of nanotubes were capable of being taken up by circulating monocytes and neutrophils in a dose-dependent manner, and less by B cells.

FACS studies were previously conducted for carbon nanotubes, mostly in the context of cancer treatment [48,49]. It was shown that tumor cells actively uptake carbon nanotubes, also when loaded with drugs. Our findings correlate with previous reports and add new information on the sensitivity of cellular interactions to nanotube loading.

## 5. Conclusions

In this study, 12 different nanotube products were synthesized composed of HiPco-SWCNTs or carboxyl-SWCNTs in combination with a NOTCH1 siRNA and/or MTX. The products were then extensively tested to determine their potential as a drug delivery system to increase the efficacy and reduce the off-target effects of MTX. In vivo studies show that HiPco-SWCNTs accumulate in arthritic joints. They were therefore further attached to MTX and siRNA. The attachment efficiencies were comparable to previously reported products. The CE% of MTX obtained for HiPco-SWCNTs ranged from 77% to 79%; for siRNA attachment efficiency was up to 97%. When incubated with human blood, SWCNTs showed interaction with monocytes, neutrophils and, less so, with B cells, in a dose-dependent manner. Notably, HiPco-SWCNTs had higher uptake efficiency, which was enhanced by the presence of siRNA. These results indicate that HiPco-SWCNTs are potent vehicles for drug delivery, with potential for use in treatment of rheumatoid arthritis.

## Figures and Tables

**Table 1:** Overview of conjugates. PEI = polyethyleneimine; EE% = encapsulation efficiency; CE% = coupling efficiency.

Name	Nanotube	PEG/EE%	PEI	RNA Attachment/Efficiency (%)	Drug/CE%
C1	HiPco-SWCNT	mPEG-DSPE, DSPE-PEG-NH_2_/52%	-	siRNA/97%	MTX/79%
C2	HiPco-SWCNT	mPEG-DSPE, DSPE-PEG-NH_2_	-	siRNA/91%	-
C3	HiPco-SWCNT	mPEG-DSPE, DSPE-PEG-NH_2_	-	sc siRNA/93%	MTX/78%
C4	HiPco-SWCNT	mPEG-DSPE, DSPE-PEG-NH_2_	-	sc siRNA/90%	-
C5	HiPco-SWCNT	mPEG-DSPE, DSPE-PEG-NH_2_	-	-	MTX/77%
C6	HiPco-SWCNT	mPEG-DSPE, DSPE-PEG-NH_2_	-	-	-
C7	Carboxyl-SWCNT	mPEG-DSPE, DSPE-PEG-NH_2_	PEI	siRNA/91%	MTX/78%
C8	Carboxyl-SWCNT	mPEG-DSPE, DSPE-PEG-NH_2_	-	siRNA/98%	-
C9	Carboxyl-SWCNT	mPEG-DSPE, DSPE-PEG-NH_2_	PEI	sc siRNA/90%	MTX/71%
C10	Carboxyl-SWCNT	mPEG-DSPE, DSPE-PEG-NH_2_	-	sc siRNA/87%	-
C11	Carboxyl-SWCNT	mPEG-DSPE, DSPE-PEG-NH_2_	-	-	MTX/83%
C12	Carboxyl-SWCNT	mPEG-DSPE, DSPE-PEG-NH_2_	-	-	-

**Table 2:** Sequences of the single strand RNA used in this study. s (sense), as (anti sense), sc (scrambled).

Name	Sequence	Purity (%)	Yield (%)
NOCH1_s	5′-r(ACUAUGCUCGUUCAACUUCCCmUmU)-3ʹ	90	10
NOCH1_as	5′-r(GGGAAGUUGAACGAGCAUAGUmUmU)-3′	94	4
sc_s	5′-r(AUGAUCCACGUUCUUUCACCCmUmU)-3′	94	5
sc_as	5′-r(GGGUGAAAGAACGUGGAUCAUmUmU)-3′	99	5

**Table 3:** Amount of intact siRNA in nanoparticles C1, C3 and control at different time points. nd = not detected.

Applied Conjugate	C1 (Amount of siRNA Released (µg)/Release%)	C3 (Amount of siRNA Released (µg)/Release%)	Control Naked siRNA (µg/Release%)
**Time Points**			
0 min	1.36 (91%)	1.28 (85%)	0.96 (64%)
30 min	1.2 (80%)	0.8 (53%)	0.8 (53%)
1 h	0.72 (48%)	0.64 (43%)	nd
2 h	0.64 (43%)	0.4 (27%)	nd
4 h	0.24 (16%)	0.16 (11%)	nd
8 h	0.16 (11%)	nd	nd

**Table 4:** Average size diameter and PDI of the HiPco-SWCNTs (obtained from SEM).

Product	Average Diameter Size/nm	PDI
Solubilized HiPco-SWCNT	343.5 ± 42.58	0.015
C1	518.8 ± 114.64	0.049
C3	407.67 ± 120.21	0.087

## Data Availability

All the data related to this study can be found in the main paper and in Supplementary Information.

## Supplementary Materials

The following are available online at https://www.mdpi.com/article/10.3390/pharmaceutics13040453/s1, Figure S1: Key steps in preparation of products C1–C12 used in this study, Figure S2: Calibration curve of methotrexate, Figure S3: Calibration curve of DSPE-PEG-NH_2_, Figure S4: MALDI MS and HPLC results for siRNA, Figure S5: Mice with highlighted arthritis and normal joint, Figure S6. Arthritis imaging of HiPco-cy5.5 on control group (healthy mice; right leg), Figure S7: Arthritis imaging of HiPco-cy5.5 on arthritic mice (right leg), Figure S8: Arthritis imaging by HiPco-cy5.5 on arthritis mice (right leg), Figure S9: Signal ratio of arthritis imaging by HiPco-cy5.5 (right leg), Figure S10: Whole body imaging of arthritis mice (circulation time: 6–12 h), Figure S11: Uptake of different nanoparticles. a. Internalization of most dextran-coated and other long-circulating nanoparticles by tissue macrophages (in lymph nodes, liver, spleen, bone marrow) and in circulating monocytes [37]. b. Nonspecific uptake of PbS nanoparticles. c. Uptake of PbS nanoparticles by different immune cells [37], Figures S12–S15: Arthritis imaging on day 3, Figures S16–S19: Arthritis imaging on day 6, Figure S20: Relative fluorescence intensity of HiPco and PbS in joint and normal tissue on day 6.
