# Supplementary material for: Carbon Nanotubes—Potent Carriers for Targeted Drug Delivery in Rheumatoid Arthritis
Source: Pharmaceutics. 2021 Mar 27;13(4):453. doi: 10.3390/pharmaceutics13040453 (PMC8066293; doi:10.3390/pharmaceutics13040453)
Supplement: Supplementary file 1 [file pharmaceutics-13-00453-s001.pdf]

pharmaceutics

Supplementary Materials: Carbon Nanotubes—Potent Carriers for Targeted Drug Delivery in Rheumatoid Arthritis

Camilla Kofoed Andersen, Sangita Khatri, Jonas Hansen, Sofie Slott, Rohith Pavan Parvathaneni, Ana C. Mendes, Ioannis S. Chronakis, Shu-Chen Hung, Narendiran Rajasekaran, Zhuoran Ma, Shoujun Zhu, Hongjie Dai, Elizabeth D. Mellins and Kira Astakhova

1. General

1.1. UV-Vis spectroscopy for concentration determination of solubilized HiPco-SWCNT and carboxyl-SWCNT.

Measurements were performed on a UV-Vis spectrophotometer at 808 nm, Infinite200PRO (TECAN, Männedorf, Switzerland). 200 μL of sample was initially loaded onto a Nunc™ MicroWell™ 96-Well Microplates. The obtained UV-Vis absorption spectra were recorded by the i-control™ Microplate Reader (TECAN, Männedorf, Switzerland).

1.2. UV-Vis spectroscopy for concentration determination of MTX

Measurements were performed on a UV-Vis spectrophotometer at 303 nm, Infinite200PRO. 200 μL of sample was initially loaded onto a Nunc™ MicroWell™ 96-Well Microplates. The obtained UV-Vis absorption spectra were recorded by the i-control™ Microplate Reader. The absorbance of unknown samples was compared to a standard curve, see Figure 1.

1.3. UV-Vis spectroscopy for concentration determination of siRNA

Measurements were performed on a QIAxpert (QIAGEN, Hulsterweg, Netherlands) at 260 nm. The obtained UV-Vis absorption spectra were analyzed and the OD260 concentration was recorded for all the analyzed samples.

1.4. RNA synthesis

Oligonucleotides were synthesized using standard (Bz-A-CE, Ac-C-CE, Ac-G-CE, U-CE) and modified (2'-O-Me-U-CE) phosphoramidites from Glen Research. Solid-phase synthesis conditions using Biosset ASM-800ET DNA/RNA Synthesizer with reagents purchased from Sigma Aldrich: TCA Deblock, DCI activator 0.25 M, Oxidizer 0.02 M, Cap A and Cap B. The phosphoramidites were all prepared in 0.07M solutions using dry acetonitrile and oligonucleotides synthesized on 1 μmol scale using universal solid support 1000 Å (CPG 1000 Å) from Sigma Aldrich. Oligonucleotides were cleaved from solid support using methylamine solution (33 wt. % in absolute ethanol) at 65 °C for 2 h, desilylated with triethylamine trihydrofluorid in dry triethylamine and dry N-methyl-2-pyrrolidone and precipitated from cold acetone. The identity of oligonucleotides was established by mass spectrometry (MS) using an Autoflex speed MALDI-TOF mass spectrometer (Bruker Daltonics, Hamburg, Germany). Samples were co-spotted with 3-Hydroxypicolinic acid as matrix on a MTP AnchorChip target plate for the analysis. The obtained mass spectra were recorded by the flexControl 3.4 (Bruker Daltonics, Germany) software. The oligonucleotides were purified on an Ultimate 3000 UHPLC (Dionex, Sunnyvale, CA, USA) using a DNA-Pac RP (Thermo Fisher Scientific, Waltham, MA, USA) column (4 μm, 3.0 × 100 mm^2) with a gradient of 5–15% buffer B in A over 30 min at 60 °C. (buffer A: 0.05 M TEAA, buffer B: 25% A in acetonitrile) Peaks were monitored at 260 nm.

1.5. Annealing of siRNA/SC siRNA

siRNA was annealed by incubating equimolar amounts of sense and antisense strand in a PBS buffer solution (100μL, 5×, pH 7.4), The suspension was incubated in a PCR tube

on a SimpliAmp™ Thermal Cycler (Thermo Fisher Scientific, Waltham, MA, USA) for 10 min at 85 °C and thereafter cooled to rt over 30 min. The concentration of annealed duplex was measured on a QIAGEN QIAxpert at 260 nm.

2. Synthesis overview

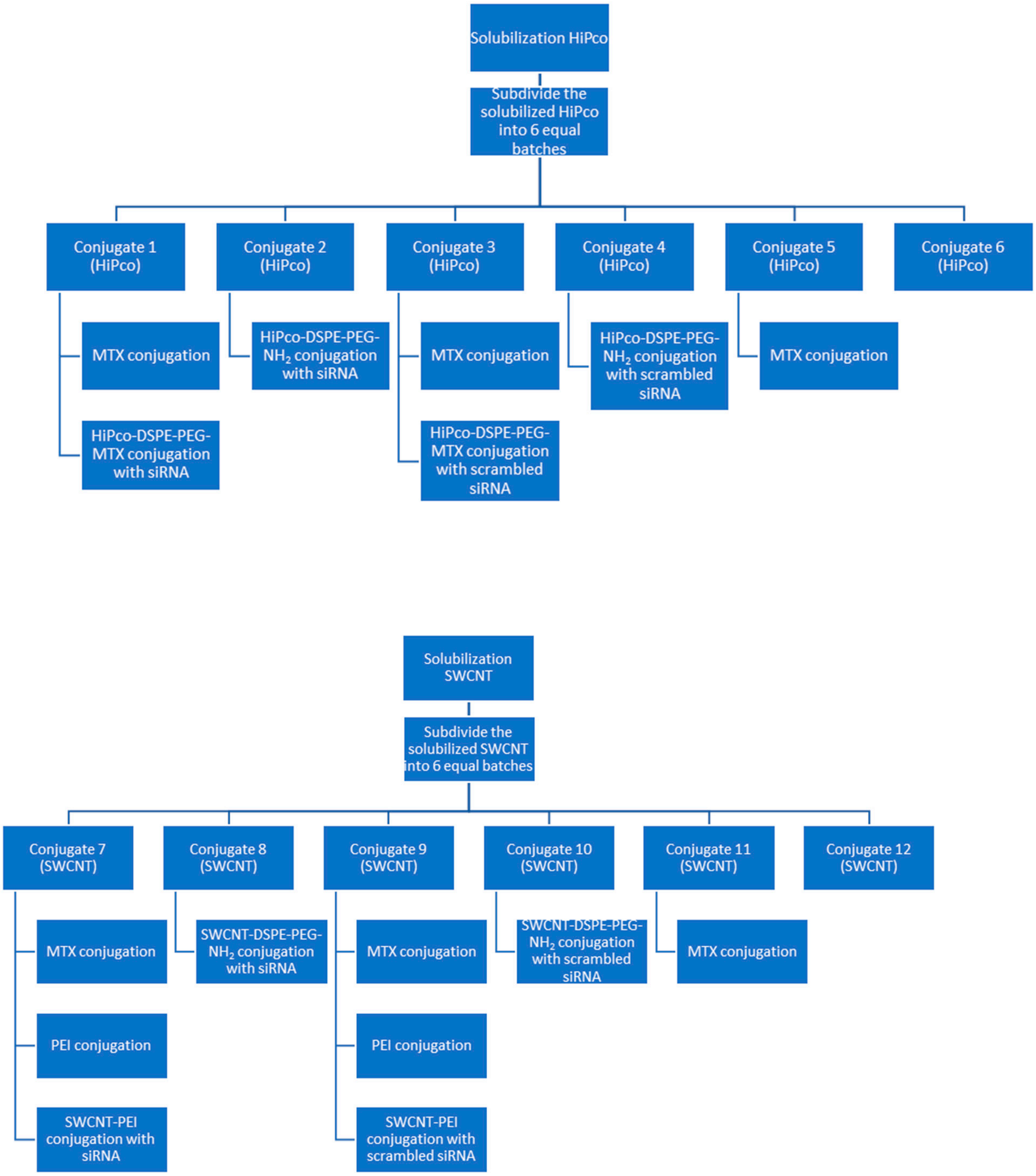

Figure S1. Key steps in preparation of conjugates C1-C12 used in this study.

3. Synthesis

3.1. Solubilization of HiPco-SWCNT (C1-C6)

HiPco nanotube (30mg, 1eq.) was dispersed in an aqueous solution of sodium cholate (15 mg/mL, 50 mL). DSPE-PEG-NH_2 (22 mg, 754 eq.) and mPEG-DSPE (53 mg, 1846 eq.) in minimal DMSO were added and the solution was sonicated for 3h at rt. The suspension was then centrifuged at 12000 rpm and 10 °C for 8 h then cooled to 4 °C. The supernatant was then carefully collected and reduced by Speedvac at 30 °C to ≈5% of the initial volume. The formulation was purified by 30kDa Amicon® Ultra-15 Centrifugal filter and recovered in 1 mL water. The amount of DSPE-PEG-NH_2 conjugated was determined by fluorescamine assay (EE% = 52% for three independent batches). The concentration of nanotubes was measured by UV-Vis (187 μM). The formulation was reduced to dryness, yield (28 mg).

3.2. Solubilization of carboxyl-SWCNTs (C7-C12)

The PEGylation of carboxyl-SWCNTs with DSPE-PEG-NH_2 and mPEG-DSPE was achieved by the same procedure as stated for the raw HiPco SWNTs with the important difference that mass ratios are used for reagents instead for molar ratios. Carboxyl-SWCNTs (20 mg, 1 eq.) was dispersed in an aqueous solution of sodium cholate (15 mg/mL, 45 mL). DSPE-PEG-NH_2 (15 mg, 0.73 eq.) and mPEG-DSPE (36 mg, 1.78 eq.) in minimal DMSO were added and the solution was sonicated for 3h at rt. The suspension was then centrifugated at 12000 rpm and 10 °C for 8 h then cooled to 4 °C. The supernatant was then carefully collected and reduced by Speedvac at 30 °C to ≈5% of the initial volume. The formulation was purified by 30 kDa Amicon® Ultra-15 Centrifugal filter and recovered in 1 mL water. The amount of DSPE-PEG-NH_2 conjugated was determined by fluorescamine assay (EE% = 63–80% for three independent batches). The concentration of nanotubes was measured by UV-Vis (122 μM). The formulation was reduced to dryness, yield (19 mg).

3.3. Coupling of MTX to HiPco SWNT-DSPE-PEG-NH_2 (C1, C3, C5)

The covalent coupling of MTX to HiPco-DSPE-PEG-NH_2 was achieved by EDC/NHS chemistry in aqueous bicarbonate buffer (10×, pH 8.4). The amount used of MTX and coupling reagents were determined based on the fluorescamine assay. NHS (1.5 mg, 13.0 μmol, 8 eq.), EDC (2.5 mg, 13.0 μmol, 8 eq.), MTX (2.5 mg, 5.5 μmol, 3.4 eq.), and 500 μL of bicarbonate buffer (10×, pH 8.4), were allowed to react for 1.5 h in the dark at rt before addition of the solubilized HiPco-DSPE-PEG-NH_2 (23 mg) containing DSPE-PEG-NH_2 (8.16 mg, 1.6 μmol, 1 eq.), and 500 μL of bicarbonate buffer (10×, pH 8.4). The mixture was stirred at rt for 3 h followed by overnight at 5 °C. The product was purified by 30 kDa Amicon filtration and the coupling efficiency was measured by quantifying the unreacted MTX (CE%: 79%, C1; 78%, C3; 77%, C5). The formulation was reduced to dryness, yield (17 mg, C1; 21 mg, C3; 18 mg, C5).

3.4. Coupling of MTX to carboxyl-SWCNT-DSPE-PEG-NH_2 (C7, C9, C11)

To as solution of SWCNT-DSPE-PEG-NH_2 (164 μL, 122 μM, 20 μmol, 1 eq.) in bicarbonate buffer (10×, pH 8.4), NHS (0.5 mg, 4.3 μmol, 0.22 eq.), EDC (0.5 mg, 2.6 μmol, 0.13 eq.) and MTX (0.5mg, 1.1μmol, 0.05eq.) was added. The ratio DSPE-PEG-NH_2:NHS:EDC:MTX was 1:1.5:0.90:0.38. Water was then added to give a total volume of 2 mL and the reaction was incubated for 17h at rt. The product was purified by 30 kDa Amicon filtration and the coupling efficiency was measured by quantifying the unreacted MTX (CE%: 78%, C7; 71%, C9; 83%, C11). The formulation was reduced to dryness, yield (28 mg, C7; 33 mg, C9; 30 mg, C11).

3.5. Coupling of PEI to carboxyl-SWCNT-DPSE-PEG-MTX (C7, C9)

To a solution of SWCNT-DSPE-PEG-MTX (60 μL, 333.47 μM, 20 μmol) in bicarbonate buffer (10×, pH 8.4), NHS (0.1 mg, 0.87 μmol, 0.04 eq.), EDC (0.1 mg, 0.52 μmol, 0.026 eq.) and PEI (0.144 mg, 0.000019 μmol, 0.0000096 eq.) was added. The molar ratio of PEI:NHS:EDC was 1:4540.8:3366.3. Water was then added to give a total volume of 1 mL and the reaction was incubated for 17 h. at rt. The product was purified by 30 kDa Amicon filtration and reduced to dryness, yield (37 mg, C7; 40 mg, C9).

3.6. siRNA/ sc siRNA encapsulation for nanotubes HiPco-DSPE-PEG-MTX (C1, C3)

Annealed siRNA (15547.6 g/mol, 19.4 mg, 1.25 μmol, 0.05 eq.) was added to a solution of HiPco-DSPE-PEG-MTX (60 μL, 418 μM, 25 mmol, 1 eq.). The mixture was incubated at rt for 30 min in the dark and 18 h at 5 °C. The product was purified by 30kDa Amicon filtration and the encapsulations efficiency (EE%) was estimated by measuring the non-conjugated siRNA by UV-V spectrophotometer QIAxpert at 260 nm. EE%: 97% C1, 93% C3.

3.7. siRNA/ sc siRNA encapsulation for nanotubes HiPco-DSPE-PEG-NH_2 (C2, C4)

Annealed siRNA (15547.6 g/mol, 19.4 mg, 1.25 μmol, 0.05 eq.) was added to a solution of HiPco-DSPE-PEG-NH_2 (134 μL, 187 μM, 25 mmol, 1 eq.). The mixture was incubated at rt for 30 min in the dark and 18 h at 5 °C. The product was purified by 30 kDa Amicon filtration and the encapsulations efficiency (EE%) was estimated by measuring the non-conjugated siRNA by UV-Vis spectrophotometer QIAxpert at 260nm. EE%: 91% C2, 90% C4.

3.8. siRNA encapsulation for nanotubes carboxyl-SWCNT-DSPE-PEG-MTX-PEI (C7, C9)

Annealed siRNA (15547.6 g/mol, 19.4 mg, 1.25 μmol, 0.05 eq.) was added to a solution of carboxyl-SWCNT-DSPE-PEG-MTX-PEI (60 μL, 333 μM, 25 mmol, 1 eq.). The mixture was incubated at rt for 30 min in the dark and 18 h at 5 °C. The product was purified by 30kDa Amicon filtration and the encapsulations efficiency (EE%) was estimated by measuring the non-conjugated siRNA by UV-Vis spectrophotometer QIAxpert at 260 nm. EE%: 91% C7, 90% C9.

3.9. siRNA encapsulation for nanotubes carboxyl-SWCNT-DSPE-PEG-NH_2 (C8, C10)

Annealed siRNA (15547.6 g/mol, 19.4 mg, 1.25 μmol, 0.05 eq.) was added to a solution of carboxyl-SWCNT-DSPE-PEG-NH_2 (164 μL, 122 μM, 25 mmol, 1 eq.). The mixture was incubated at rt for 30 min in the dark and 18 h at 5 °C. The product was purified by 30 kDa Amicon filtration and the encapsulations efficiency (EE%) was estimated by measuring the non-conjugated siRNA by UV-VIS spectrophotometer QIAxpert at 260 nm. EE%: 98% C8, 87% C10.

4. UV-Vis data

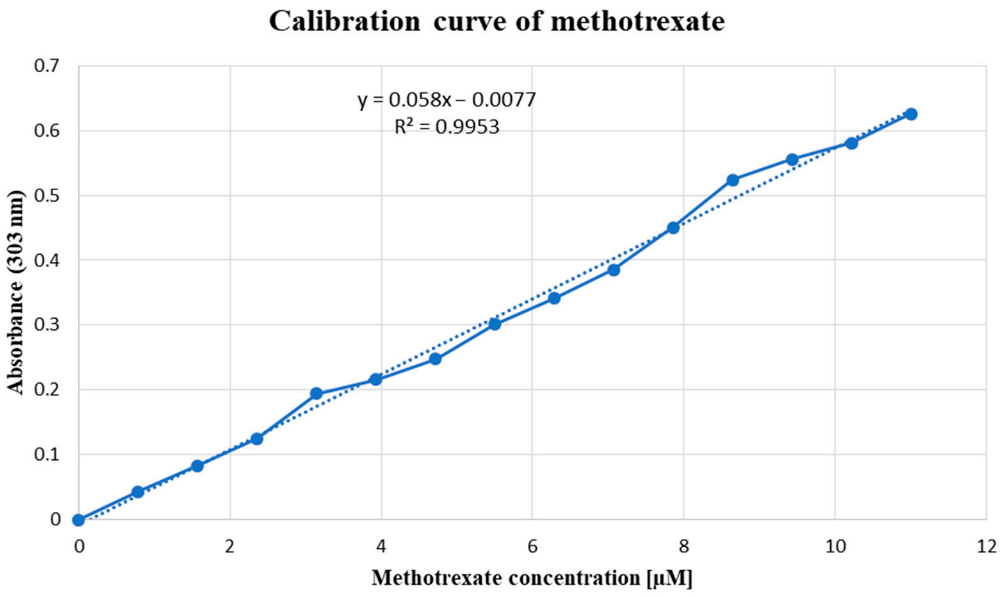

Figure S2. Calibration curve of methotrexate.

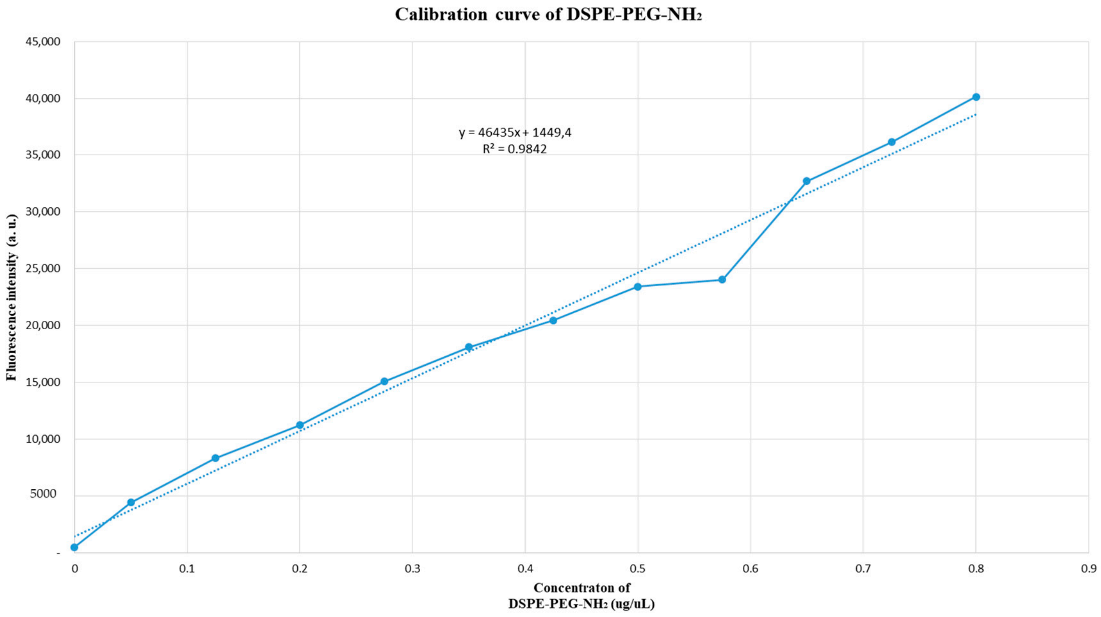

Figure S3. Calibration curve of DSPE-PEG-NH_2.

5. MALDI and HPLC characterization

Name	Sequence	MS calc. [g/mol]	MS obs. [m/z]
NOCH1_s	5'-r(AC-UAUGCUCGUUCAACU-UCCCmUmU)-3'	7170.3	7170.387
NOCH1_as	5'-r(GGGAAGUUGAAC-GAGCAUAGUmUmU)-3'	7479.6	7479.507
NOCH1_sc_s	5'-r(AUGAUCCAC-GUUCUUUCACCCmUmU)-3'	7170.3	7170.541
NOCH1_sc_as	5'-r(GGGUGAAAGAAC-GUGGAUCAUmUmU)-3'	7479.6	7479.559

MALDI NOCH1_s

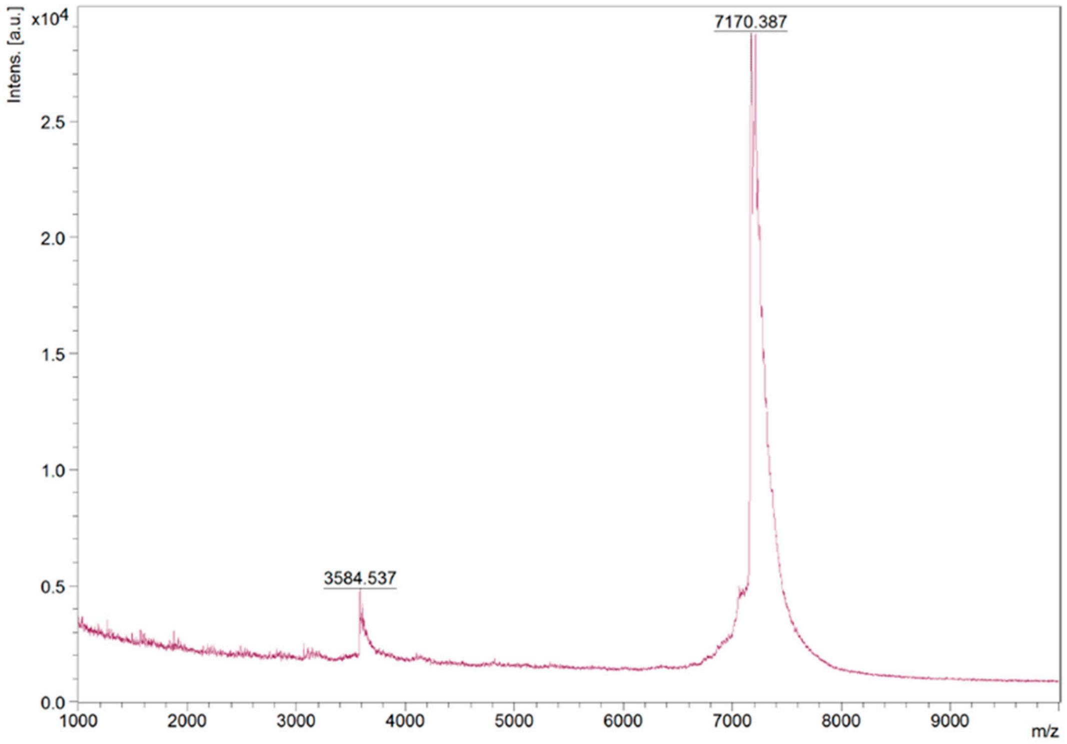

NOCH1_as

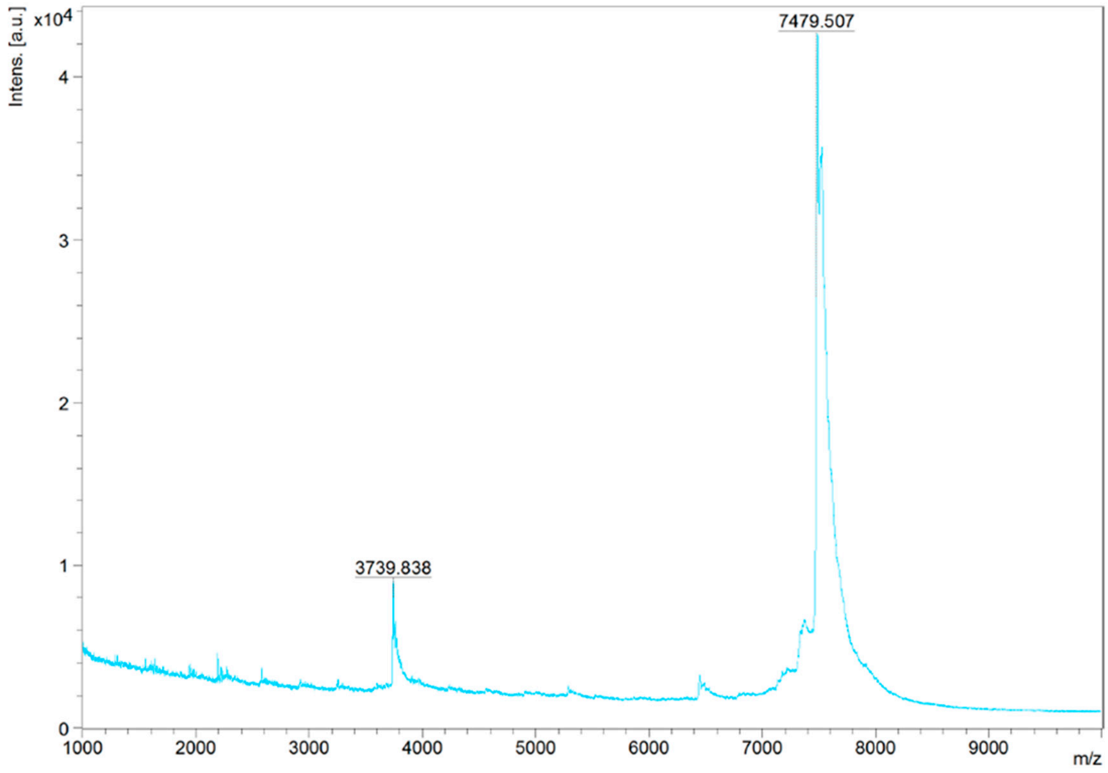

NOCH1_as_s

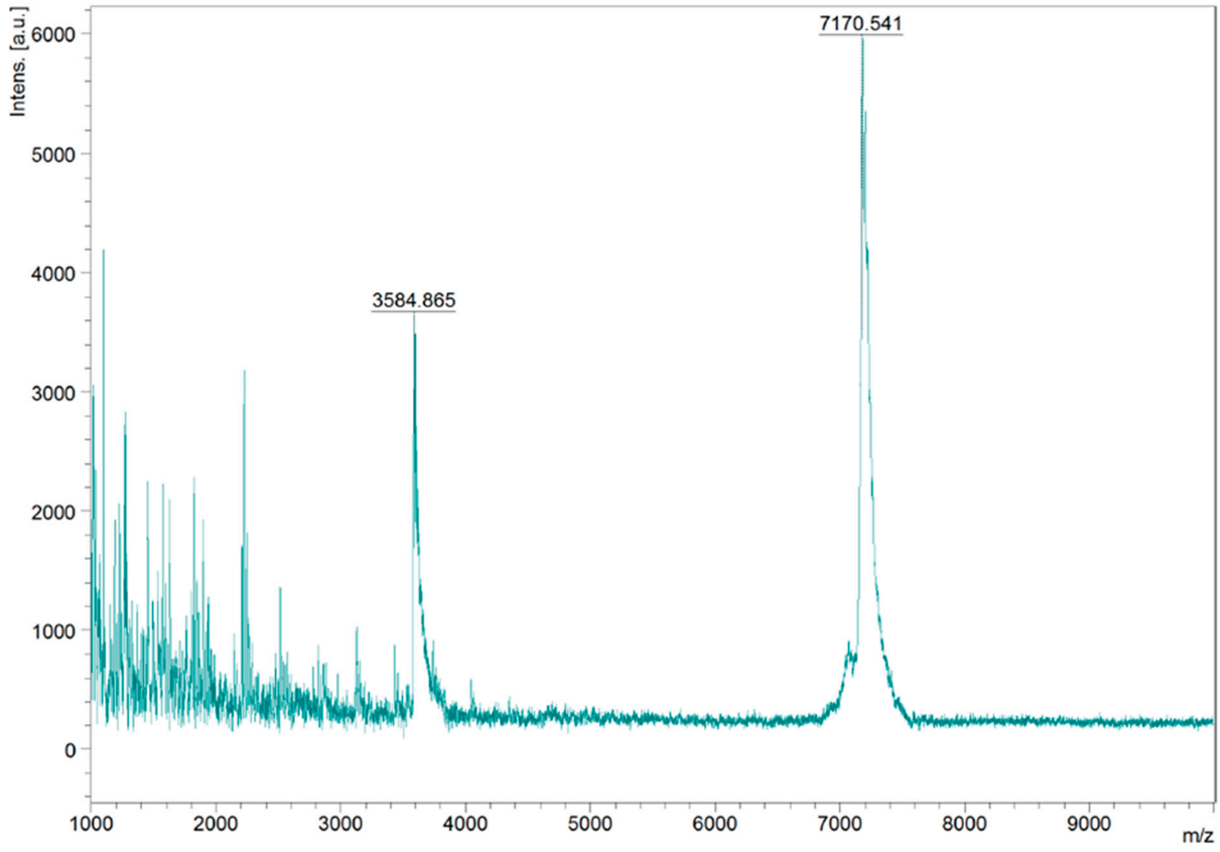

NOCH1_sc_as

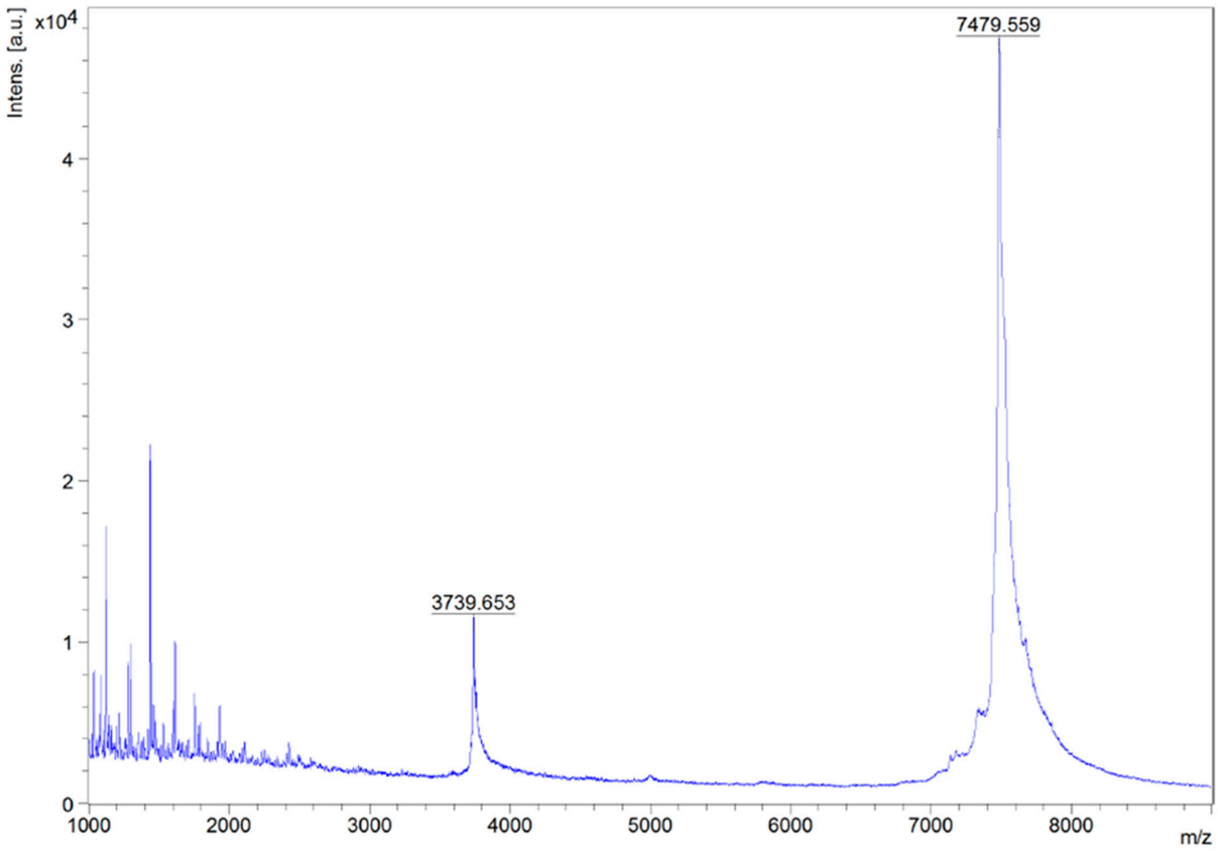
Intens. [a.u.]
x10^4
7479.559
3739.653
m/z

HPLC

NOCH1_s

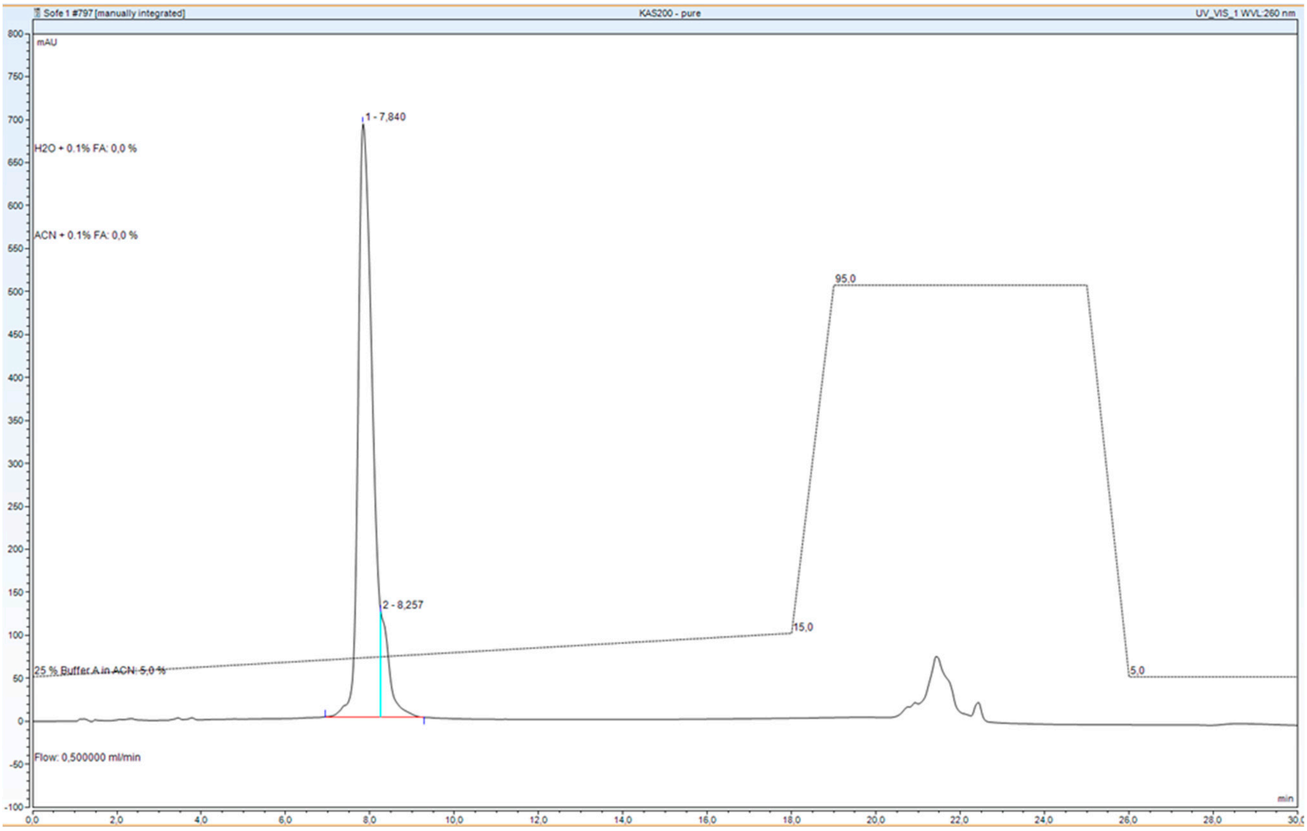
Sofe 1 #797 [manually integrated]
KAS200 - pure
UV_VIS_1 WVL:260 nm
mAU
1 - 7,840
2 - 8,257
H2O + 0.1% FA: 0,0 %
ACN + 0.1% FA: 0,0 %
95,0
15,0
5,0
25 % Buffer A in ACN: 5,0 %
Flow: 0,500000 ml/min
min

NOCH1_as

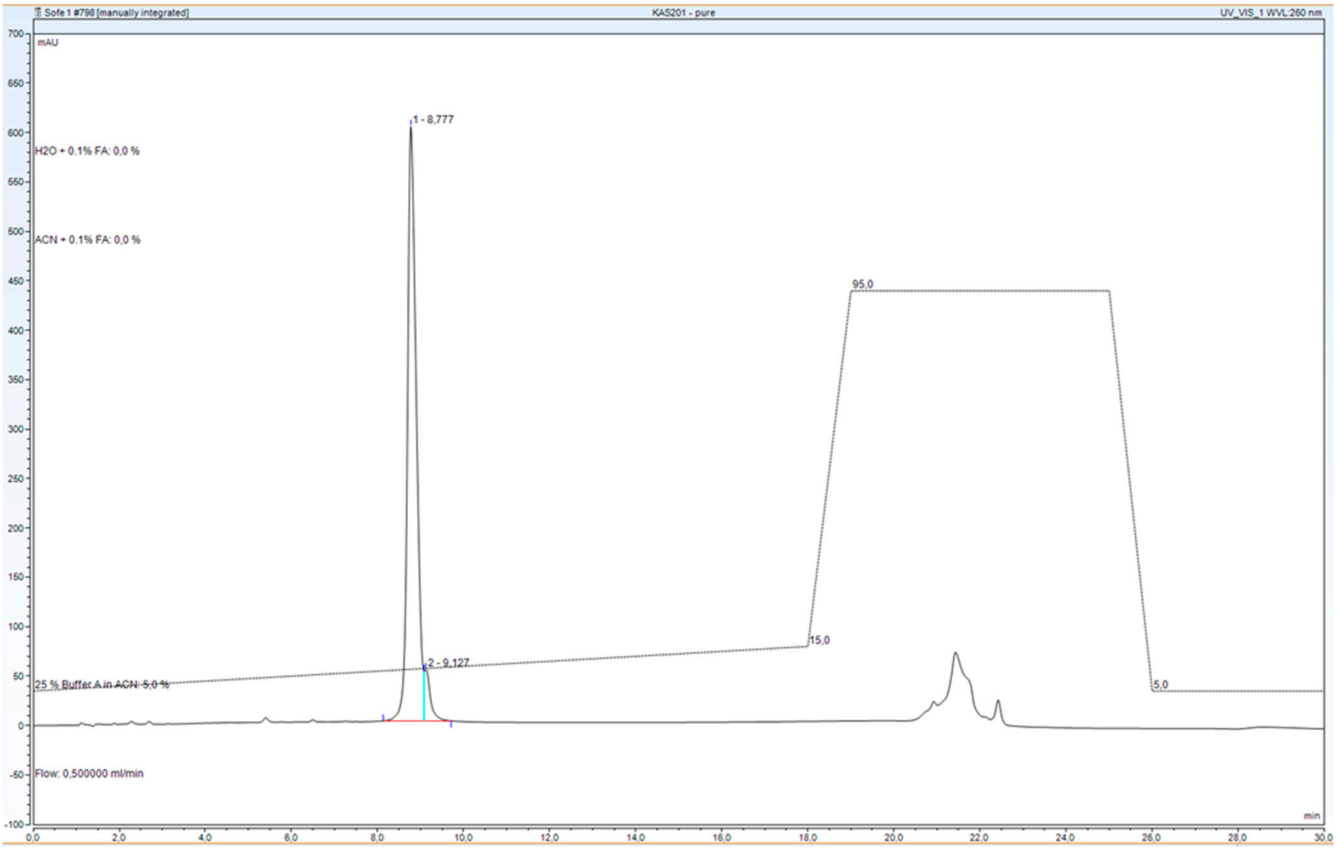

NOCH1_sc_s

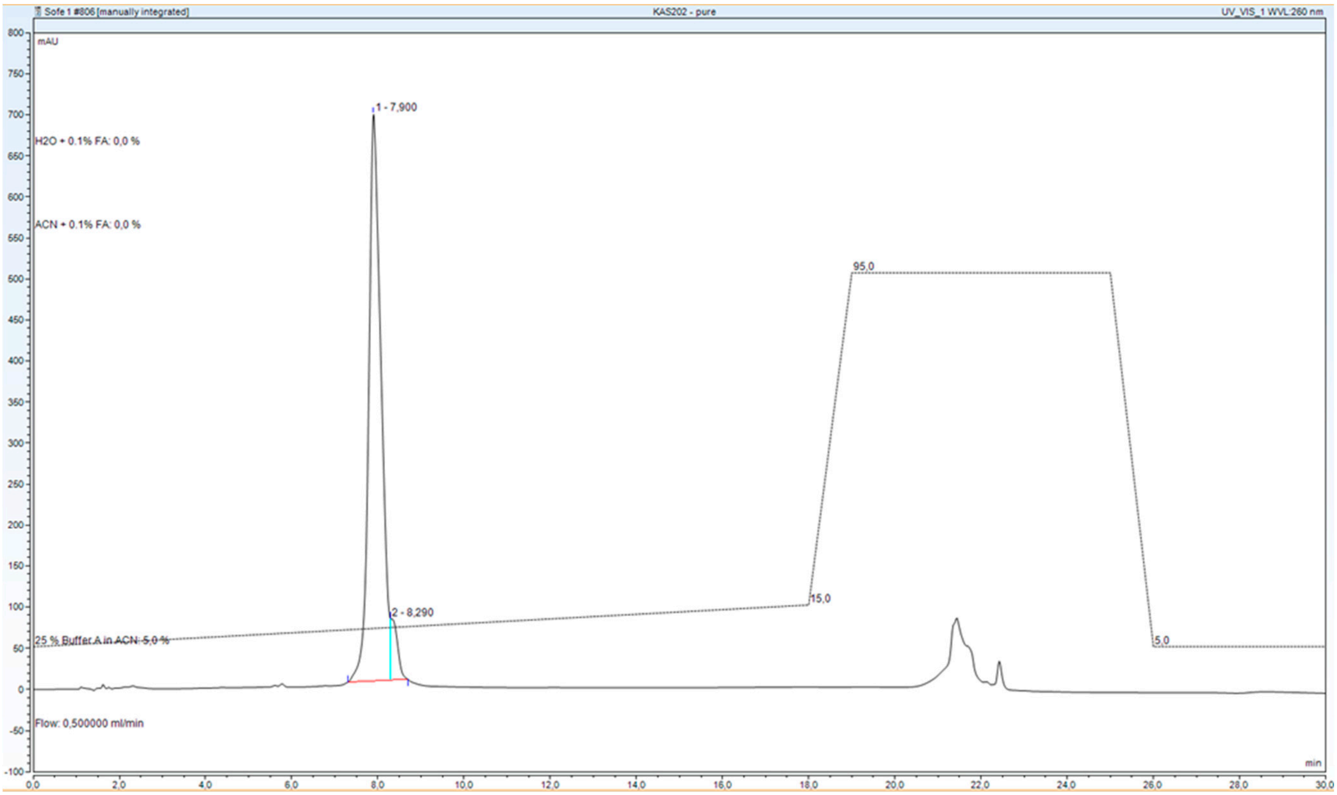

NOCH1_sc_as

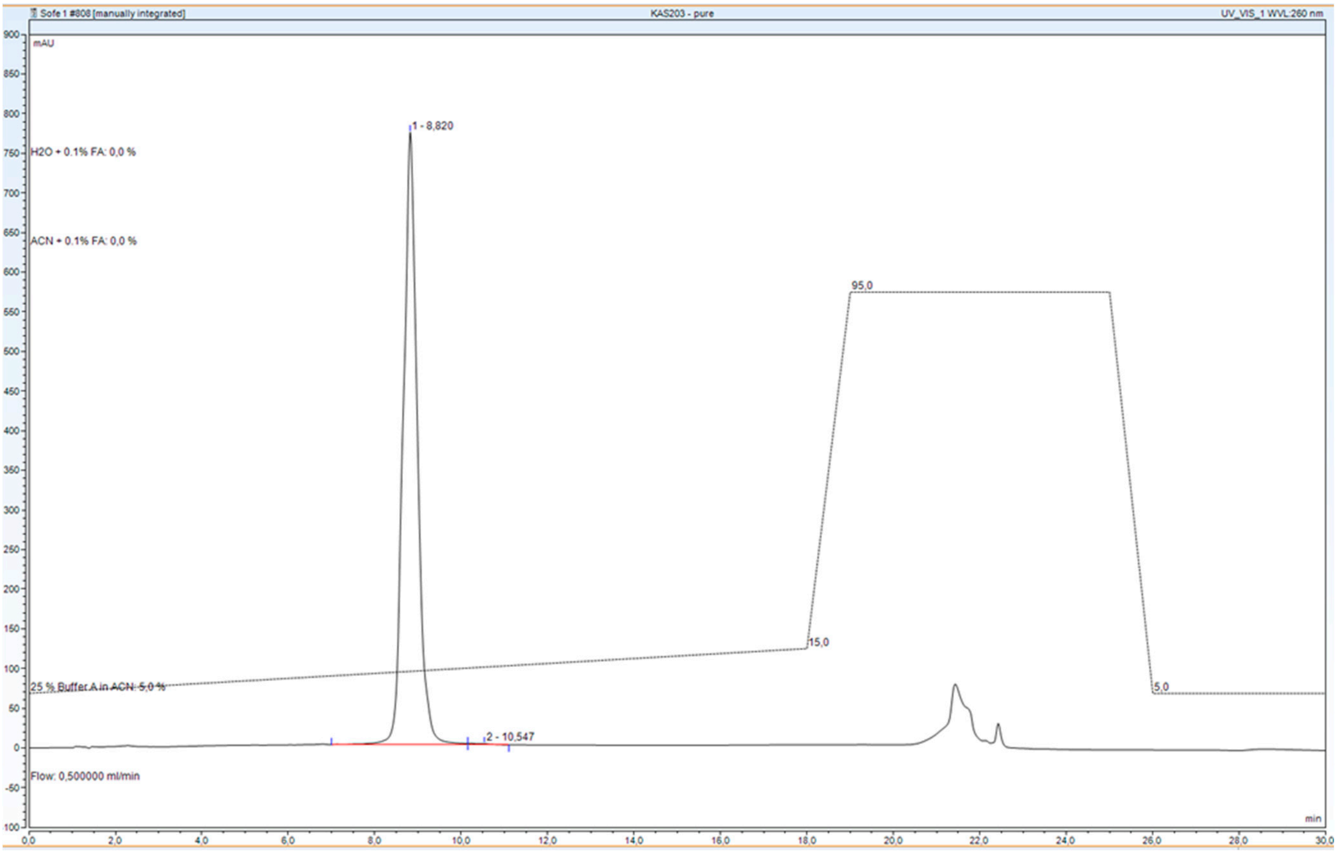

6. In vivo study—imaging results

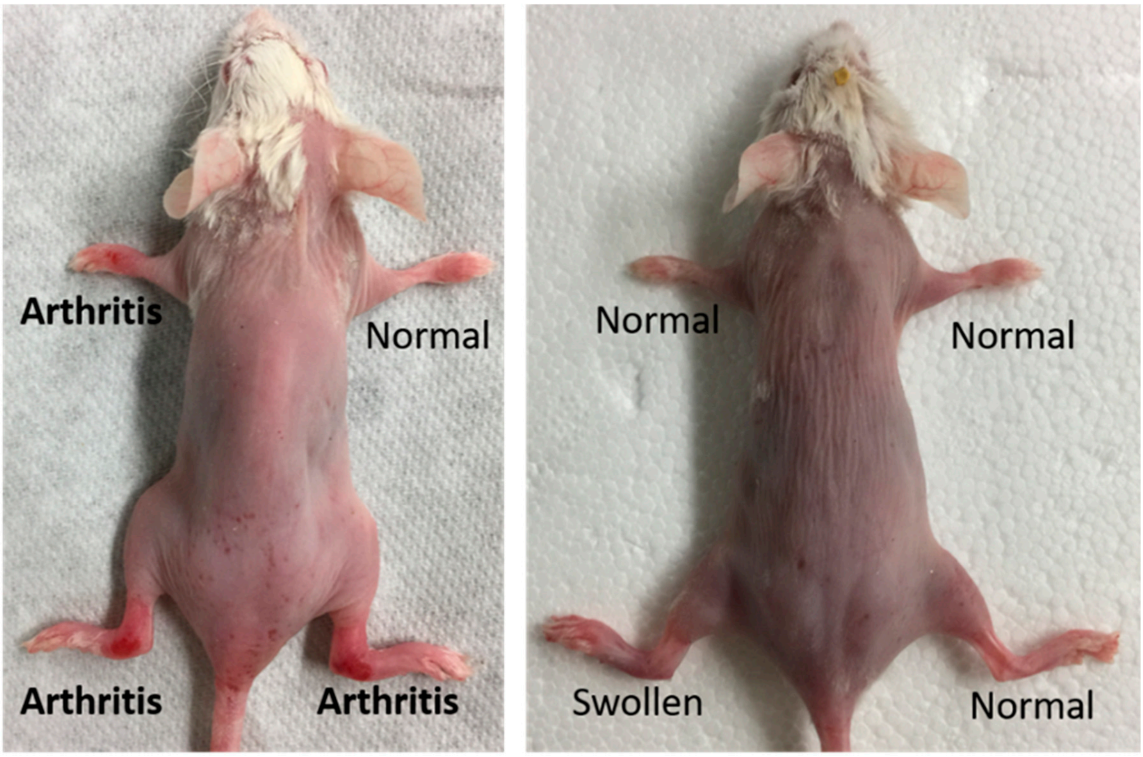

Figure S5. Mice with highlighted arthritis and normal joint.

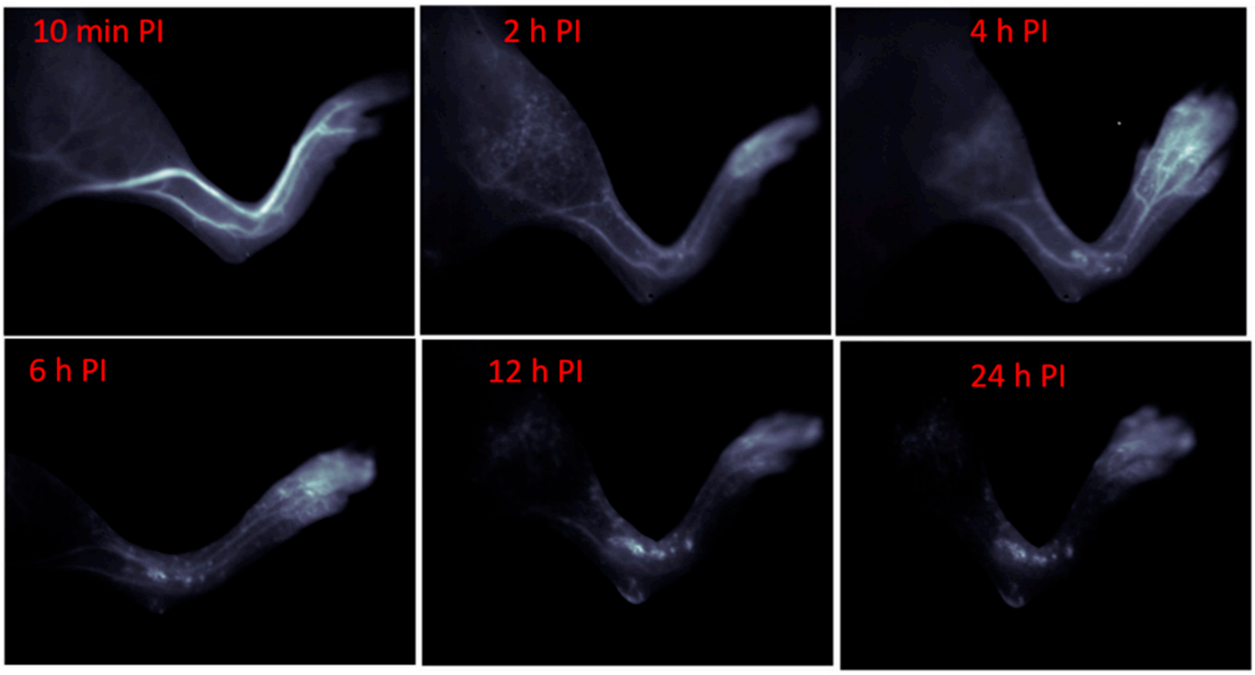

Figure S6. Arthritis imaging of HiPco-cy5.5 on control group (healthy mice; right leg). Dose (HiPco-cy5.5): 1.56 μM, OD=12.4, 200 μL. 1200 nm LP, 200 ms exposure time.

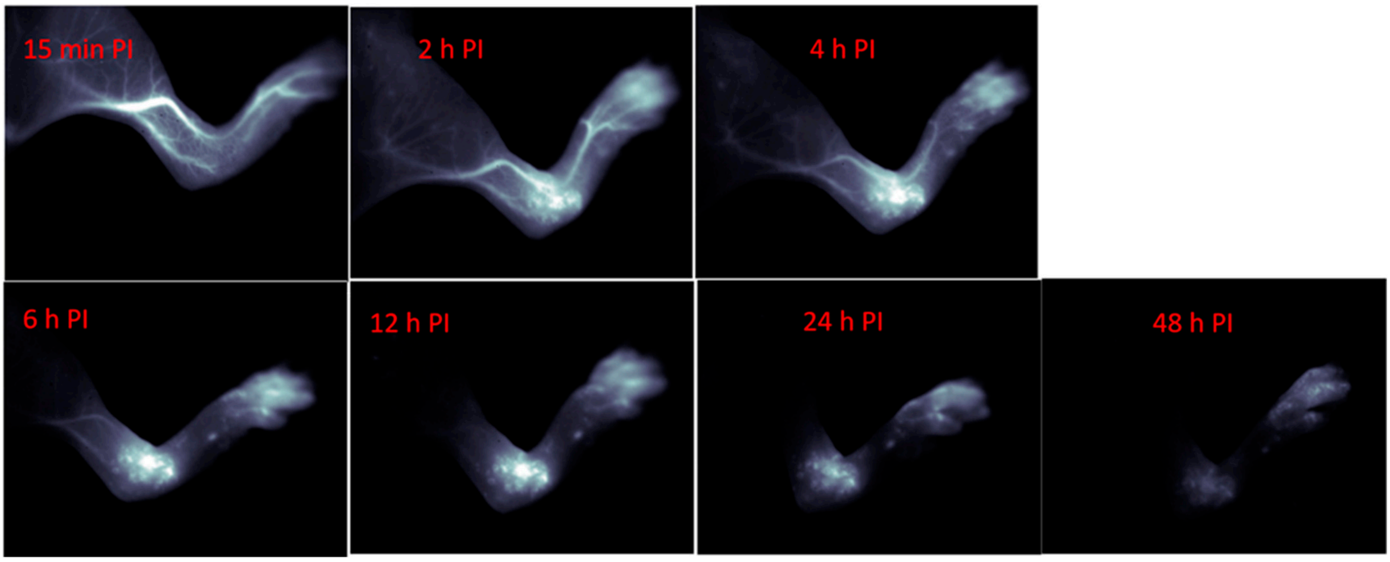

Figure S7. Arthritis imaging of HiPco-cy5.5 on arthritic mice (right leg). Dose (HiPco-cy5.5): 1.56 μM, OD=12.4, 200 μL. 1200 nm LP, 200 ms exposure time.

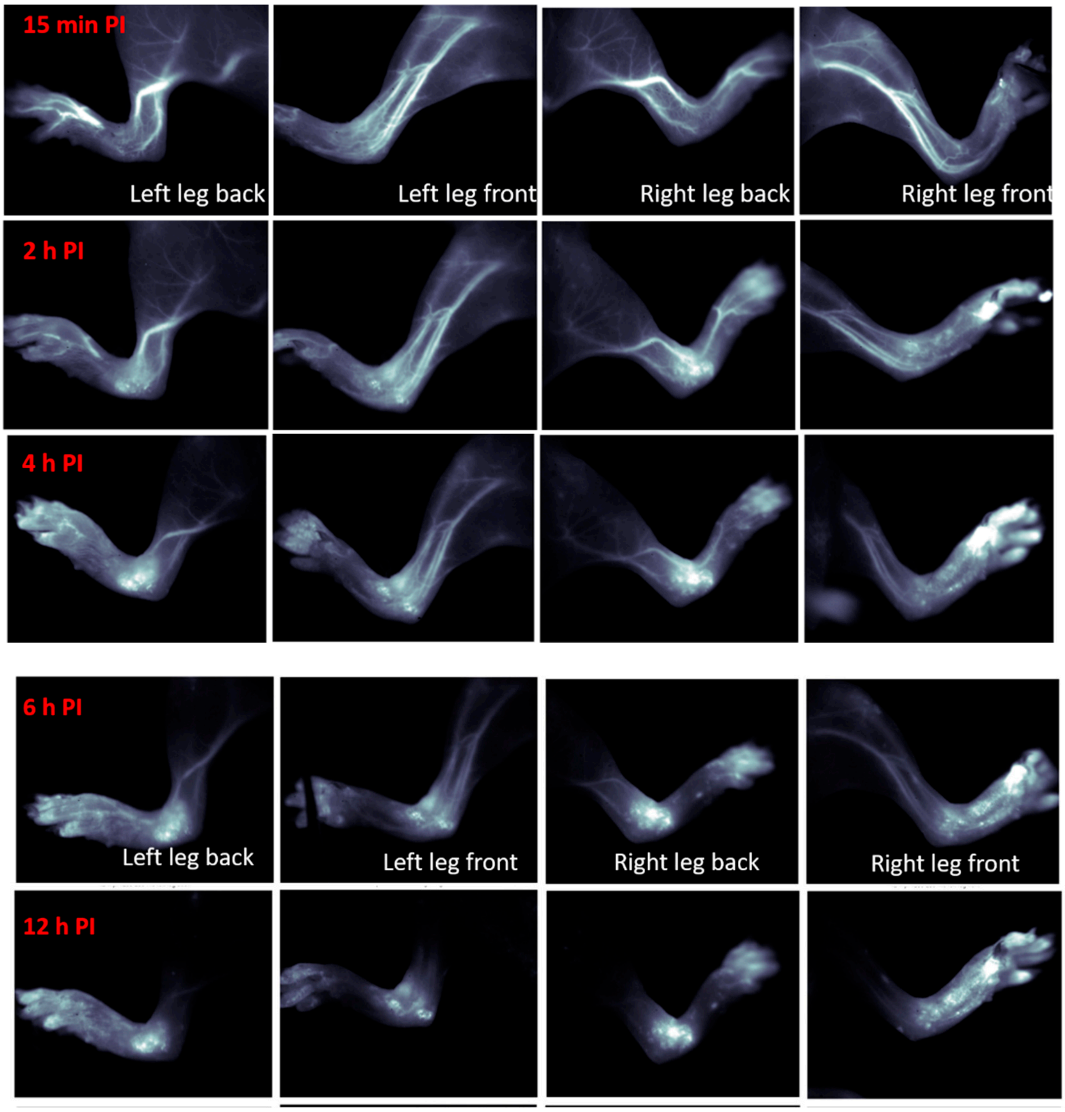

Figure S8. Arthritis imaging by HiPco-cy5.5 on arthritis mice (right leg).

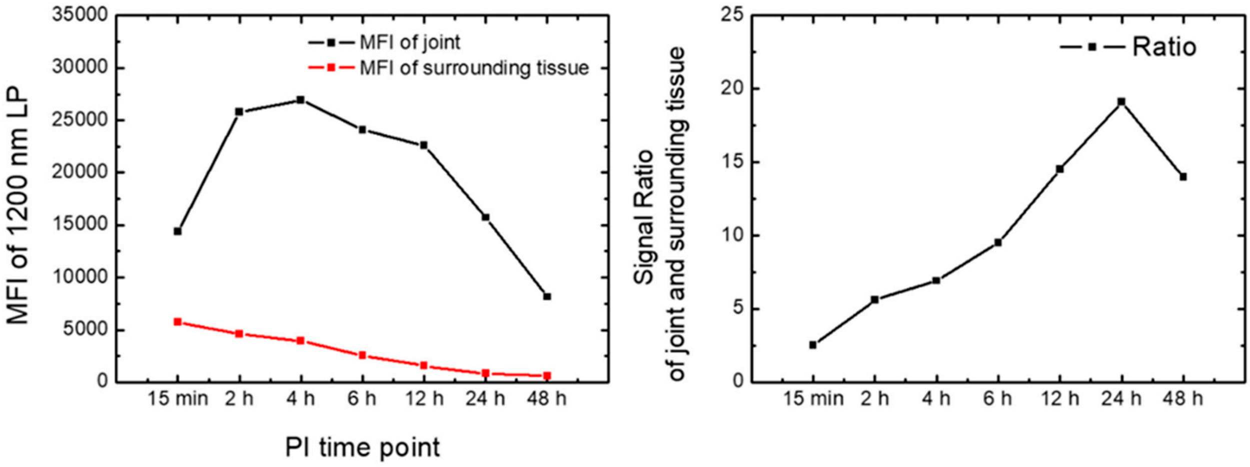

Figure S9. Signal ratio of arthritis imaging by HiPco-cy5.5 (right leg).

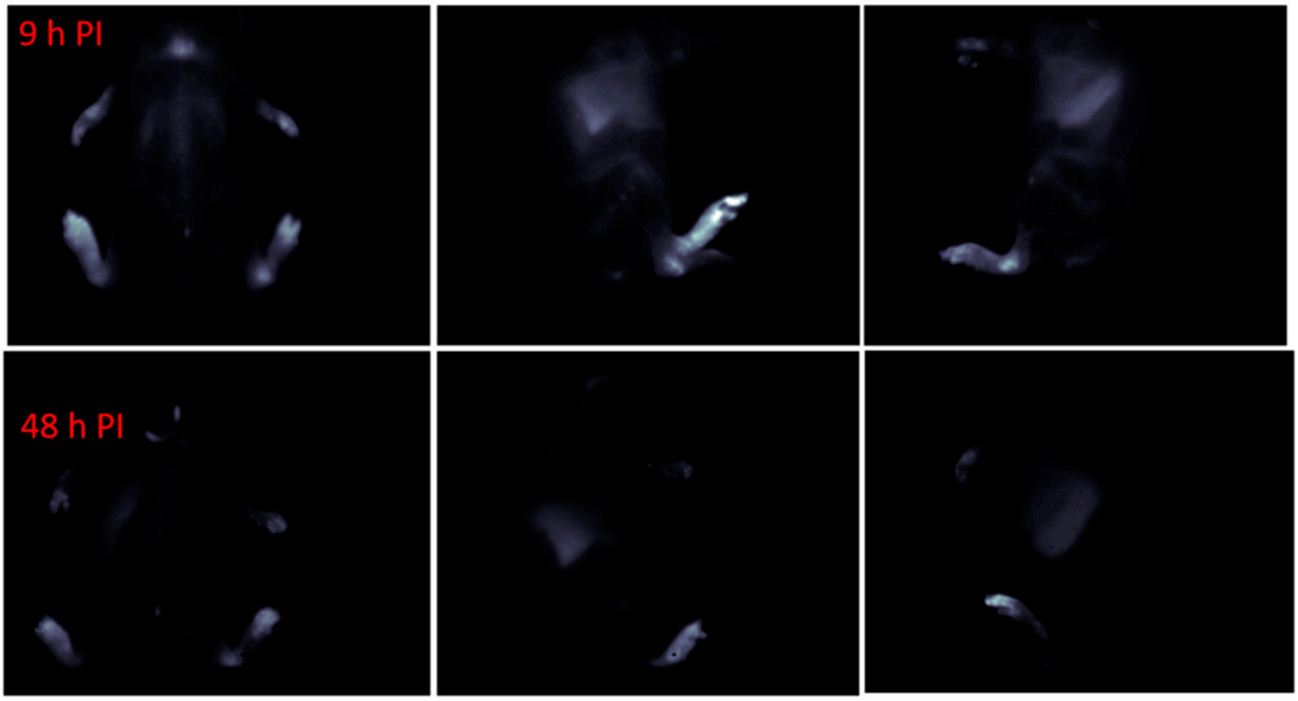

Figure S10. Whole body imaging of arthritis mice (circulation time: 6–12 h). HiPco-cy5.5 on arthritic mice, 9 h PI (1200 nm LP, 200 ms).

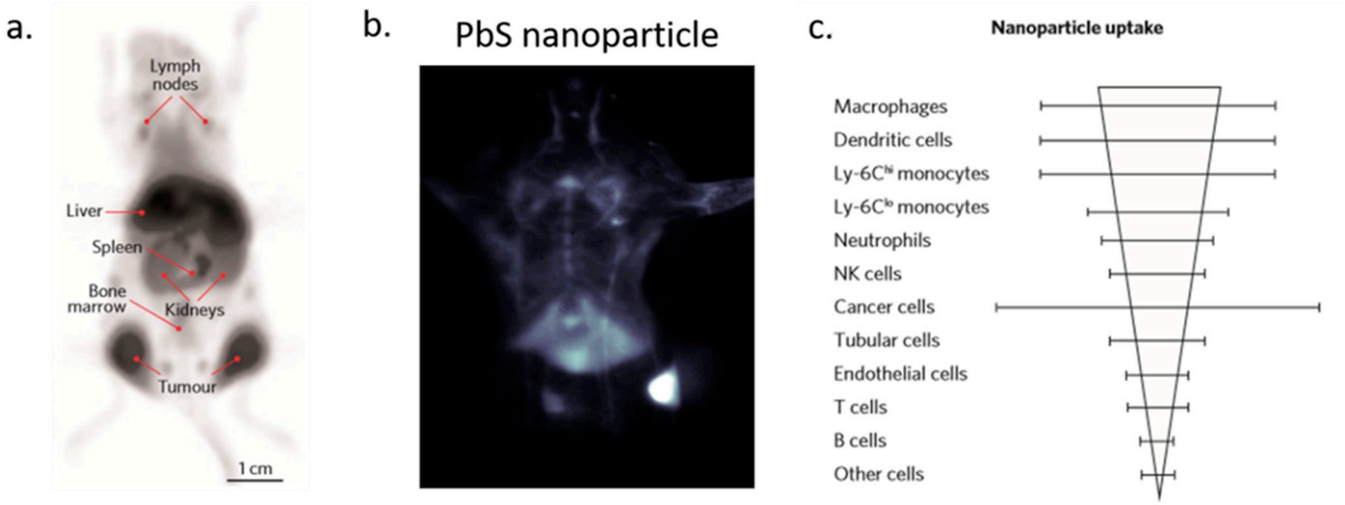

Figure S11. Uptake of different nanoparticles. (a). Internalization of most dextran-coated and other long-circulating nanoparticles by tissue macrophages (in lymph nodes, liver, spleen, bone marrow) and in circulating monocytes. (b). Non-specific uptake of PbS nanoparticles. (c). Uptake of PbS nanoparticles by different immune cells.

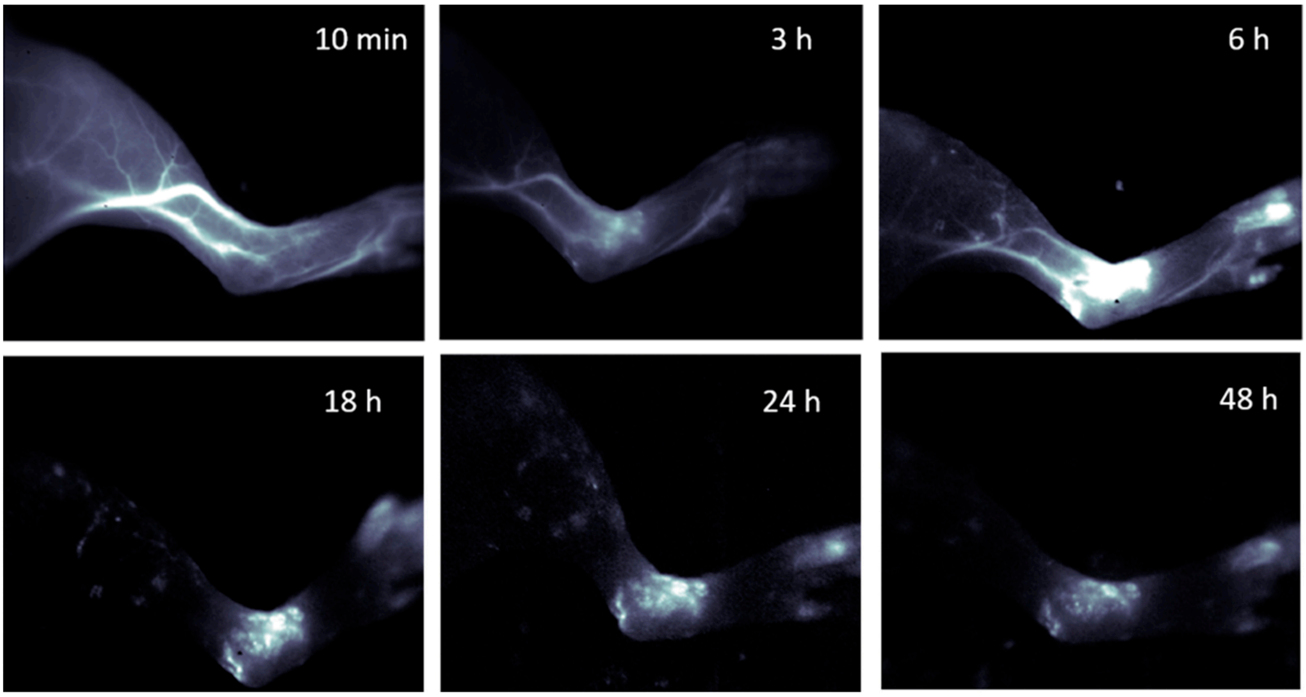

Figure S12. Arthritis imaging in Day 3. HiPco 100ms 1200LP OD=12.4, 200 μL. Scale: 10 min and 3 h– [500,40000]; others– [500,10000].

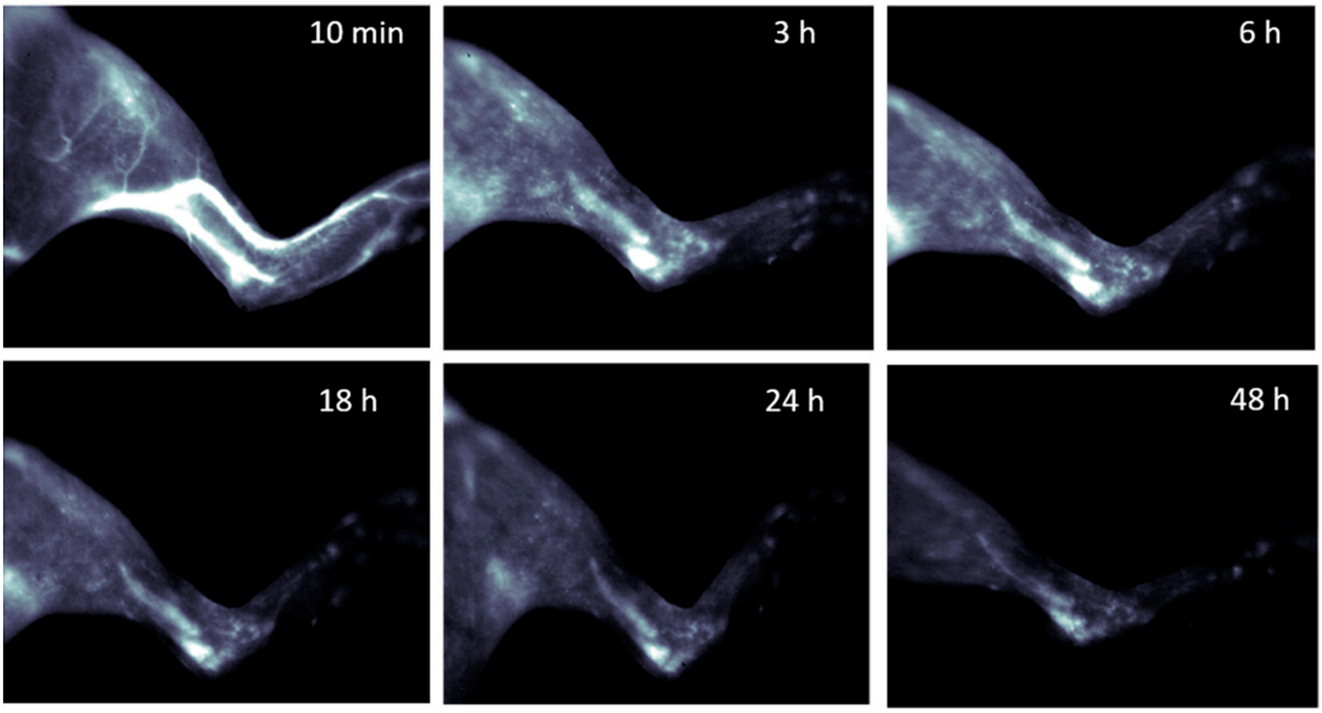

Figure S13. Arthritis imaging in Day 3. PbS 50 ms 1500LP OD=2, 200 μL Scale: [1000,12000].

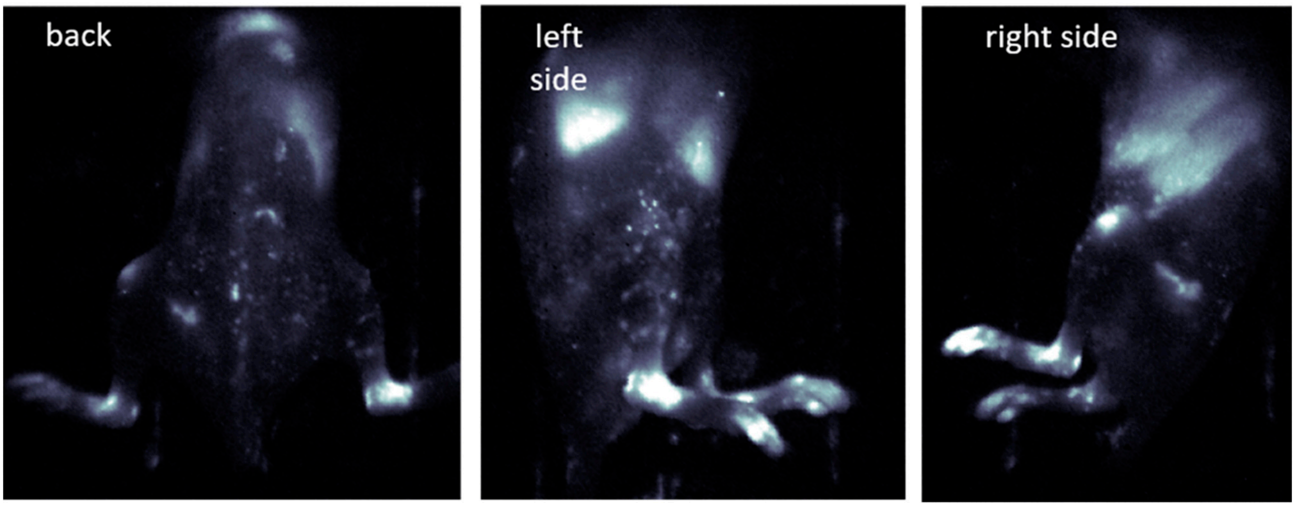

Figure S14. Whole-body images after 24 hours post injection in Day 3. HiPco 1200LP.

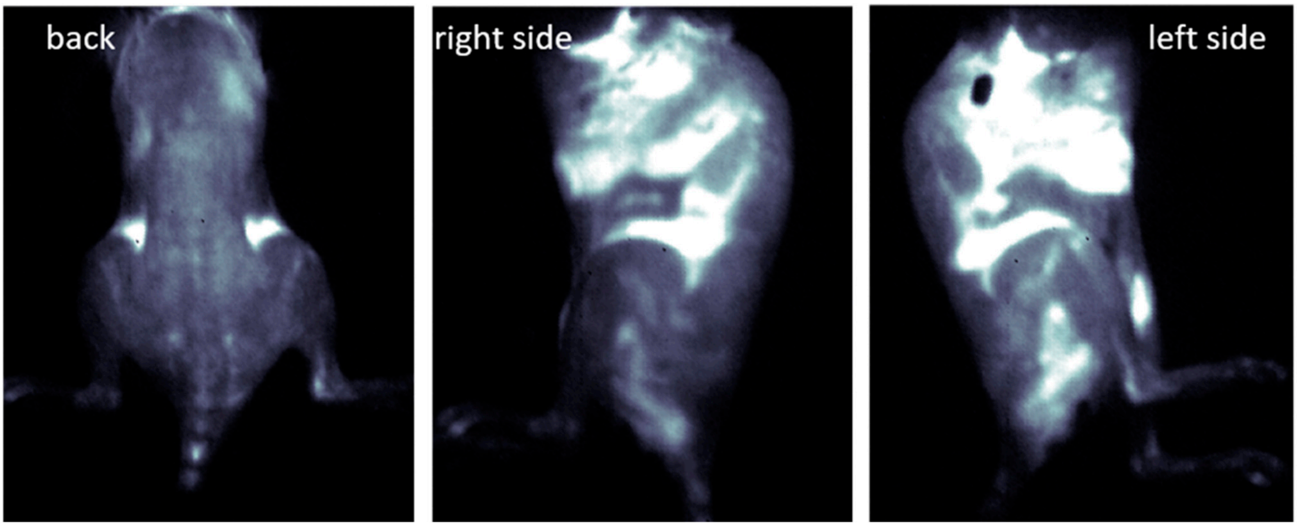

Figure S15. Day 3 Whole-body images 24 h post injection of PbS 1500LP.

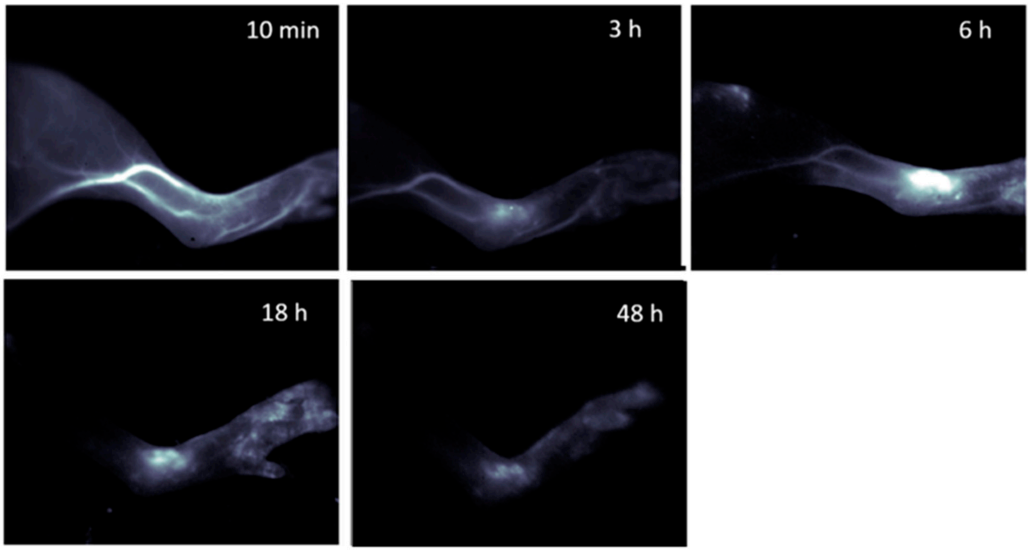

Figure S16. Arthritis imaging at Day 6. HiPco 100 ms 1200LP OD=12.4, 200 μL Scale: 10 min and 3 h– [500,30000]; others– [500,10000].

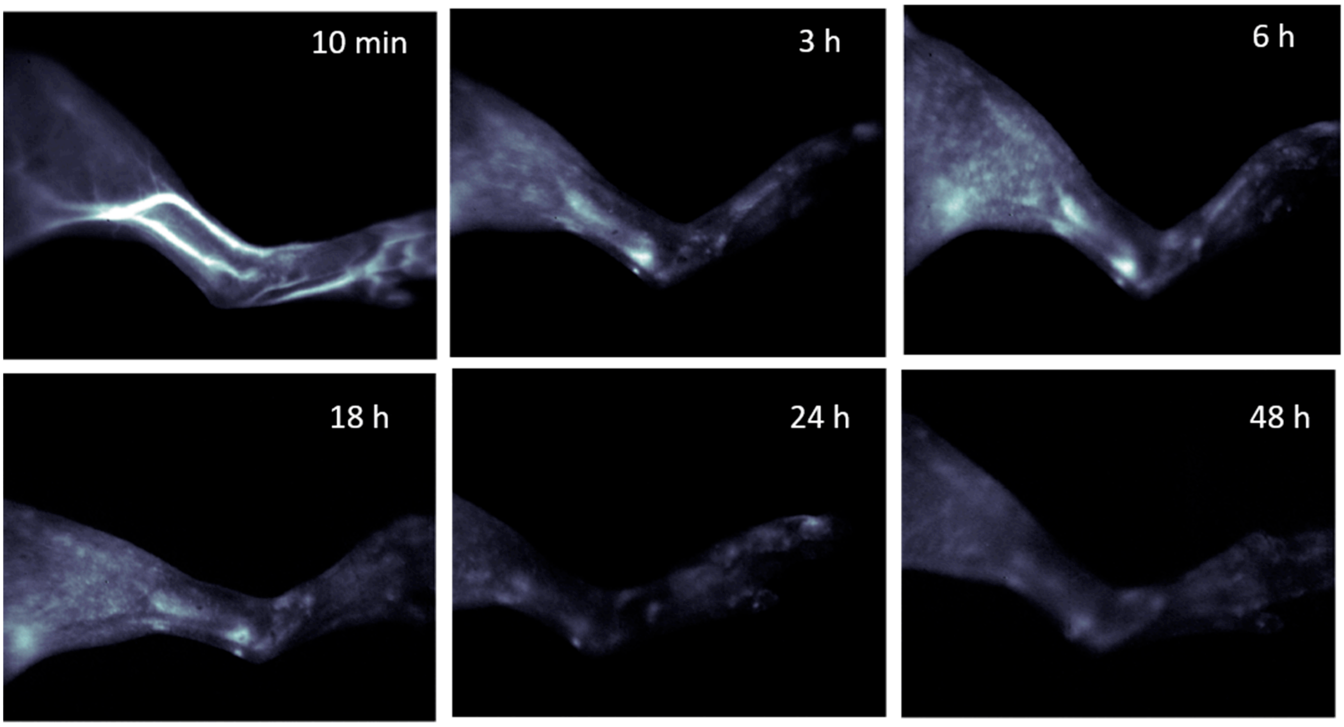

Figure S17. Arthritis imaging at Day 6. PbS 50 ms 1500LP OD=2, 200 μL Scale: 10 min [0,20000], others [0,10000].

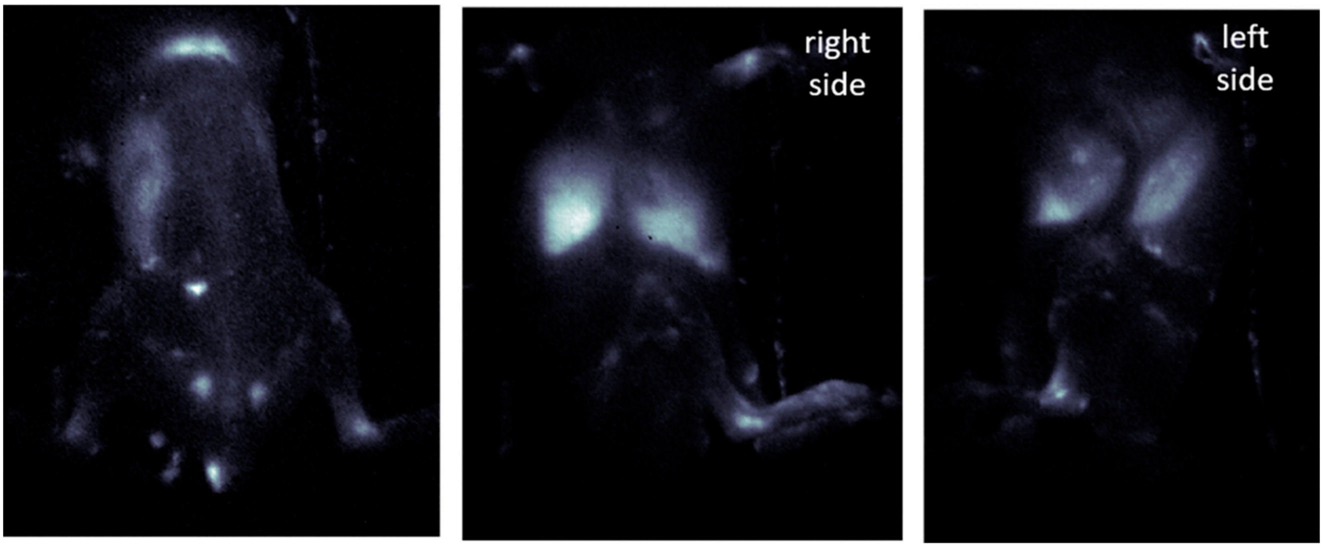

Figure S18. Arthritis imaging at Day 6. Whole-body images after 24 h post injection of HiPco 1200LP.

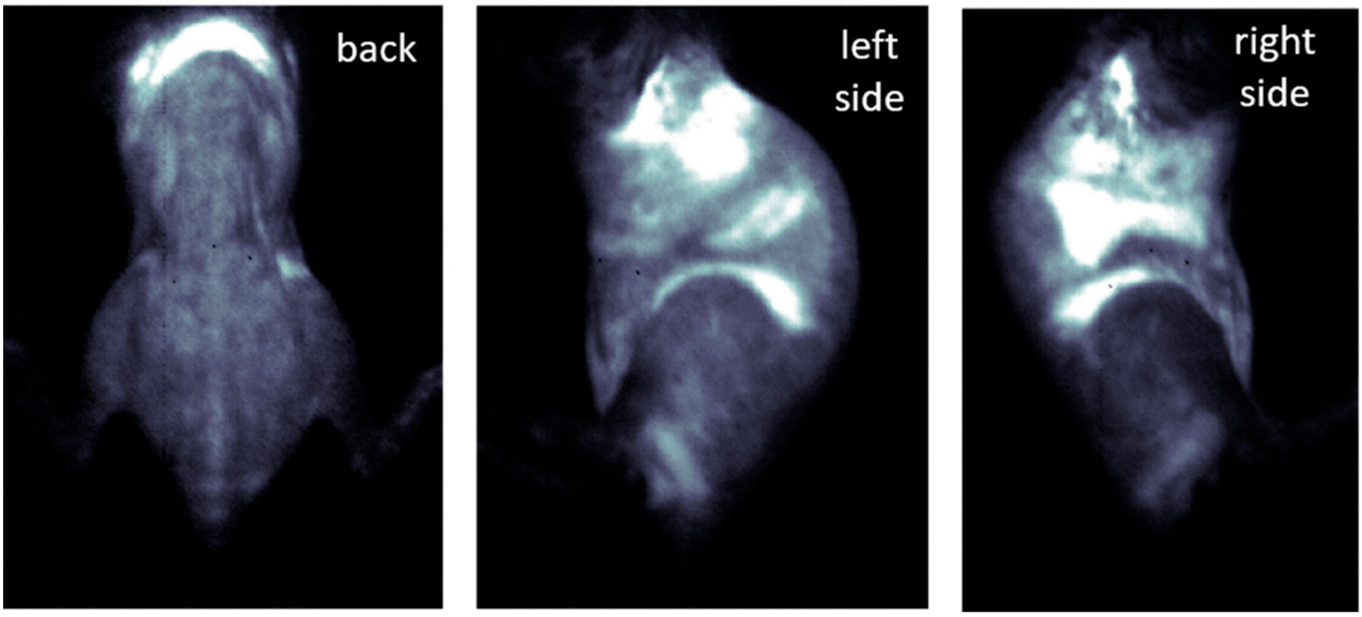

Figure S19. Arthritis imaging at Day 6. Whole-body images 24 h post injection of PbS 1500LP.

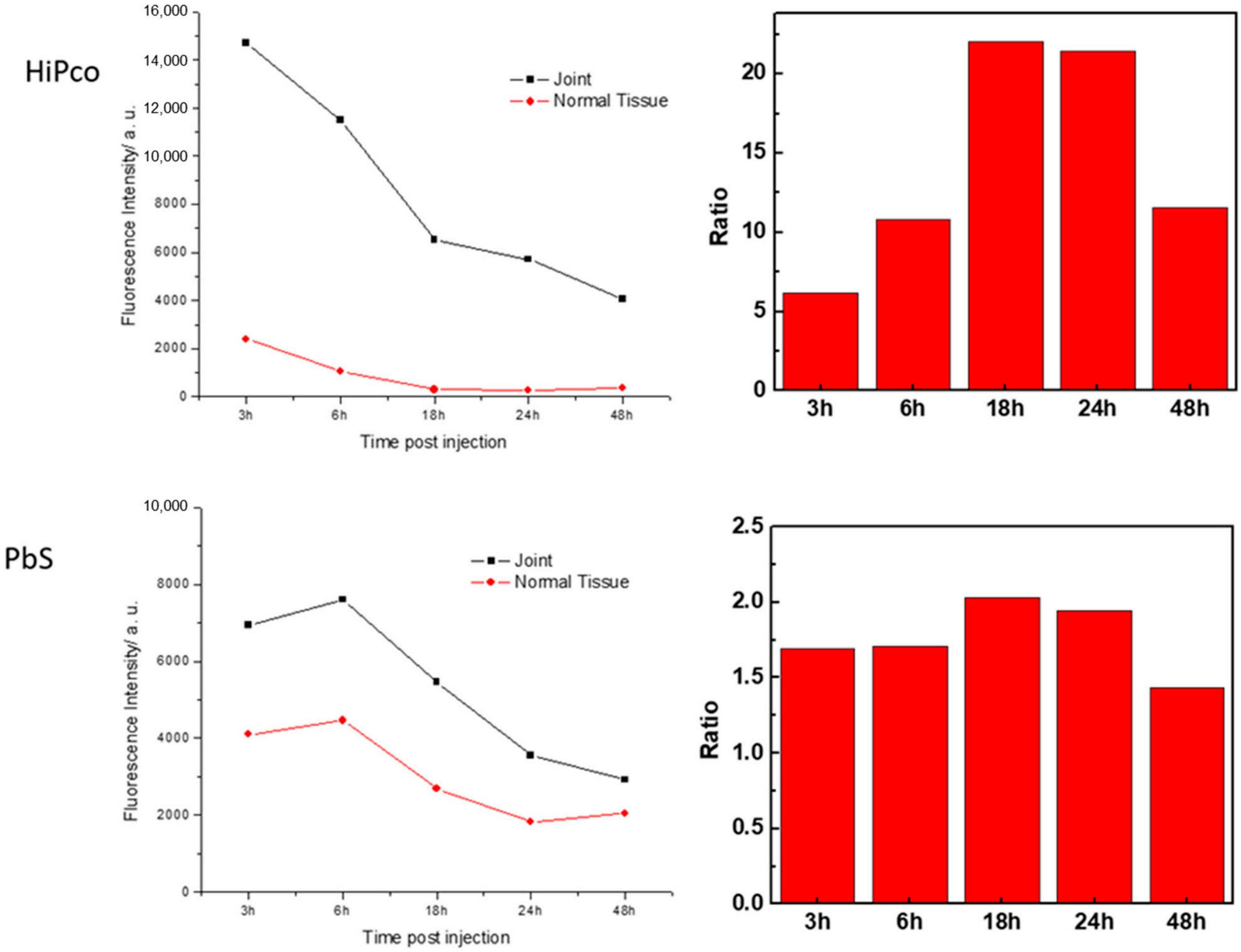

Figure S20. Relative fluorescence intensity of HiPco and PbS in joint and normal tissue in Day 6.
